# Shentong Zhuyu Decoction Alleviates Neuropathic Pain in Mice by Inhibiting the NMDAR-2B Receptor-Mediated CaMKII/CREB Signaling Pathway in GABAergic Neurons of the Interpeduncular Nucleus

**DOI:** 10.3390/ph18101456

**Published:** 2025-09-28

**Authors:** Ying Liu, Rujie Li, Haojie Cheng, Yuxin Wang, Jian Sun, Meiyu Zhang

**Affiliations:** Experimental Research Center, China Academy of Chinese Medical Sciences, Beijing 100700, China; 15156996510@163.com (Y.L.);

**Keywords:** neuropathic pain, Shentong Zhuyu Decoction, network pharmacology, NMDAR-2B/CaMKII/CREB, GABAergic neurons, spared nerve injury

## Abstract

**Background**: Shentong Zhuyu Decoction (STZYD) is a traditional Chinese medicine formula that has shown promise in alleviating neuropathic pain (NPP), yet its central mechanisms remain unclear. **Methods**: We investigated the STZYD effects on NPP using network pharmacology, in vivo assays, and analytical chemistry, focusing on molecular pathways and GABAergic neuronal modulation. **Results**: Network pharmacology revealed 254 potential STZYD targets enriched in calcium signaling and GABAergic synapse pathways, especially the NMDAR-2B/CaMKII/CREB axis. High-dose STZYD (1.25 g·mL^−1^) and ifenprodil (6 mg·kg^−1^) reversed hyperalgesia and anxiety-like behaviors in spared nerve injury (SNI) mice, and microdialysis showed that STZYD and ifenprodil reduced the glutamate, D-serine, aspartate, glycine, and gamma-aminobutyric acid levels in the interpeduncular nucleus (IPN). Immunofluorescence and fiber photometry showed reduced c-Fos expression and suppressed GCaMP signals in IPN GABAergic neurons, with chemogenetic experiments confirming their role in pain modulation. Multimodal molecular biology experiments demonstrated that STZYD and ifenprodil significantly downregulated the GluN2B, p-CaMKII, and p-CREB expressions within the IPN. We identified 145 constituents in STZYD through high-resolution mass spectrometry analysis, among which 40 were absorbed into plasma and 7 were able to cross the blood–brain barrier and accumulate in the IPN. Molecular docking revealed the strong binding of licoricesaponin K2 and senkyunolide F to NMDAR-2B. **Conclusions**: STZYD exerts dose-dependent antinociceptive effects by modulating IPN GABAergic neuronal activity through the inhibition of the NMDAR-2B-mediated CaMKII/CREB pathway.

## 1. Introduction

Neuropathic pain (NPP) is a chronic pain syndrome resulting from lesions or diseases affecting the somatosensory nervous system that impacts 6.9–10% of the general population [1]. The defining characteristics of NPP include spontaneous pain, hyperalgesia (an exaggerated pain response to noxious stimuli), and allodynia (the perception of pain from normally non-painful stimuli) [2]. As a major global public health challenge, NPP not only causes physical dysfunction but also contributes to comorbid psychological conditions, such as depression and anxiety, significantly impairing the quality of life and work capacity of patients [3,4]. Current clinical management commonly includes tricyclic antidepressants (TCAs), serotonin–norepinephrine reuptake inhibitors (SNRIs), gabapentinoids, opioids, topical agents, and botulinum toxin type A [5,6]. However, these pharmacotherapies are limited by dose-dependent adverse effects, such as somnolence, constipation, xerostomia, dizziness, weight gain, and localized reactions [7].

The interpeduncular nucleus (IPN), located in the midbrain, plays a critical role in emotional control, pain modulation, reward processing, and learning/memory [8,9,10]. The IPN is dominated by GABAergic neurons that receive glutamatergic input from the medial habenula (MHb) [11,12], and its activity is enhanced in rodent models of chronic constriction injury [13] and sleep-deprivation-induced hyperalgesia [14]. The IPN bidirectionally modulates pain perception and the affective state through reciprocal connections with the medial prefrontal cortex (mPFC), septum, hypothalamus, locus coeruleus, and nucleus incertus [15,16]. These neural networks facilitate the bidirectional transmission of reward signals and aversive information processing, potentially modulating both pain perception and spatial encoding.

Shentong Zhuyu Decoction (STZYD), a classical formula documented in the Qing Dynasty (1636–1912 CE) text Yilin Gaicuo (Correcting Errors in Medical Classics) [17,18,19], has been historically used to treat pain associated with “Bi” syndrome (painful obstruction syndrome). This polyherbal formulation comprises twelve botanical constituents. Modern pharmacological studies demonstrate that STZYD alleviates neuropathic pain through the modulation of the spinal cord MAPK pathway [20]. Emerging evidence has identified specific bioactive components within STZYD, such as lumbrokinase (derived from *Pheretima aspergillum* polypeptides), *Notopterygium incisum* (Qianghuo), and paeoniflorin, as critical contributors to its analgesic efficacy [18,20]. Mechanistically, these compounds exhibit multitarget actions, such as the suppression of COX-2-mediated neuroinflammation, the regulation of TRPV1/Ca^2+^ signaling, and the enhancement of GABAergic inhibitory neurotransmission. Currently, network pharmacology is being widely applied to investigate the mechanisms of traditional Chinese medicine (TCM) formulae and is regarded as an effective tool for elucidating the complex relationships between bioactive compounds, molecular targets, and therapeutic effects [21,22]. Previous studies have predicted the potential targets of STZYD in neuropathic pain models, including the spinal cords of rats, nucleus pulposus cells, and cancer-induced bone pain [20]. However, the central nervous system (CNS) targets of STZYD have not yet been explored. In this study, we employed network pharmacology to construct a multilayered “drug–compound–target–disease” network, thereby providing a holistic and systematic perspective on the interactions between the formula and the human body.

## 2. Results

### 2.1. Network Pharmacology Analysis of STZYD

**Figure 1:**
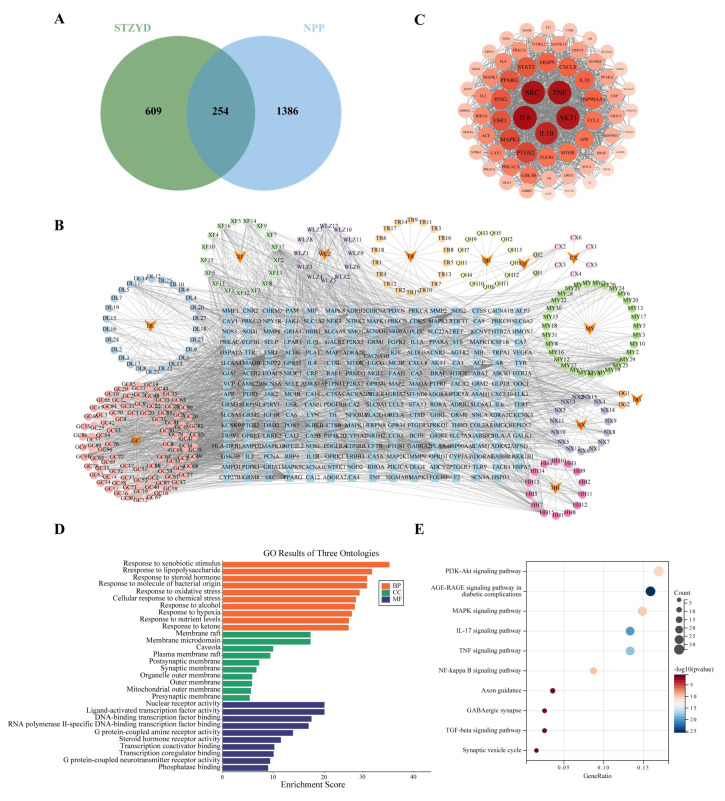
Network pharmacology prediction of Shentong Zhuyu Decoction (STZYD) in the treatment of NPP. (**A**) Venn diagram showing the overlap between STZYD and NPP. (**B**) Compound–target–pathway–disease network. Inverted triangles represent the herbal components, circles represent the active-ingredient targets, and squares represent disease nodes. (**C**) Protein–protein interaction (PPI) network of candidate targets obtained from the STRING database and visualized using Cytoscape 3.7.2. (**D**) Top 30 enriched Gene Ontology (GO) terms of STZYD candidate targets, including biological processes (BPs), cellular components (CCs), and molecular functions (MFs). (**E**) Top 10 enriched KEGG pathways of STZYD candidate targets. The x-axis represents the enrichment ratio, the circle sizes indicate the numbers of enriched genes, the y-axis denotes the KEGG pathways, and the different colors represent the adjusted *p* values.

Based on the search results from the TCMSP database, a total of 189 active ingredients were screened according to the criteria of OB ≥ 30% and DL ≥ 0.18. A total of 863 drug targets were ultimately identified using the UniProt database for standardization and matching. A search for “Neuropathic Pain” in the database yielded 1640 targets associated with NPP. By intersecting the NPP disease targets with those related to STZYD, 254 potential therapeutic targets for STZYD in the treatment of NPP were obtained. A Venn diagram illustrating this result is shown in Figure 1A. The active-ingredient–target–disease network of traditional Chinese medicine was constructed using Cytoscape 3.9.1. In Figure 1B, the nodes representing key targets (such as TNF, IL-6, AKT1, SRC, STAT3, etc.) exhibit larger sizes and denser connections, indicating that these targets function as central hubs with the highest “degree values”. These hubs are simultaneously modulated by multiple herbs and are primarily involved in the inflammatory–immune–pain pathways, including PI3K-Akt, MAPK, NF-κB, TNF, and IL-17. By importing the 254 intersecting targets into the STRING database, a protein–protein interaction (PPI) analysis was conducted, and a PPI network diagram was generated (Figure 1C). Notably, SRC, TNF, IL-1β, AKT1, IL-6, and STAT3 appeared prominently, suggesting their pivotal roles in the network and their importance as key bridges connecting other nodes within the network. GO enrichment analysis revealed significant BPs associated with blood circulation, the lipopolysaccharide response, and the regulation of cell migration, the MAPK signaling cascade, and the apoptosis signaling pathway (Figure 1D). These findings suggest that STZYD may modulate immune responses, inflammatory processes, and cell survival pathways in the treatment of NPP. The CC enrichment analysis indicated that key targets were primarily localized in membrane rafts, plasma membrane protein complexes, and receptor complexes, suggesting that STZYD may influence cellular signaling and neuronal function (Figure 1D). MF analysis highlighted cytokine activity, receptor activity, and protein kinase binding, further supporting the involvement of STZYD in inflammation, cell signaling, and gene regulation (Figure 1D). KEGG enrichment analysis highlighted the PI3K-Akt, MAPK, NF-κB, TNF, and IL-17 signaling pathways, together with the AGE-RAGE pathway linked to diabetic complications. Neuromodulatory pathways, including the TGF-β signaling, axon guidance, GABAergic synapse, and synaptic vesicle cycle pathways, were also significantly enriched (Figure 1E). Mechanistically, STZYD may bind to membrane-proximal receptors to influence downstream signaling, enhance the synaptic GABAergic tone and vesicle cycling, and regulate neurotransmitter release, thereby reducing neuronal excitability and exerting neuroprotective and analgesic effects. Taken together, within the central nervous system, STZYD may initiate GPCR- or nuclear receptor-mediated signaling cascades at membrane rafts or synaptic sites, ultimately reducing neuronal excitability and conferring neuroprotection and/or analgesia.

### 2.2. STZYD Effects on Pain Behaviors in SNI Mice

**Figure 2:**
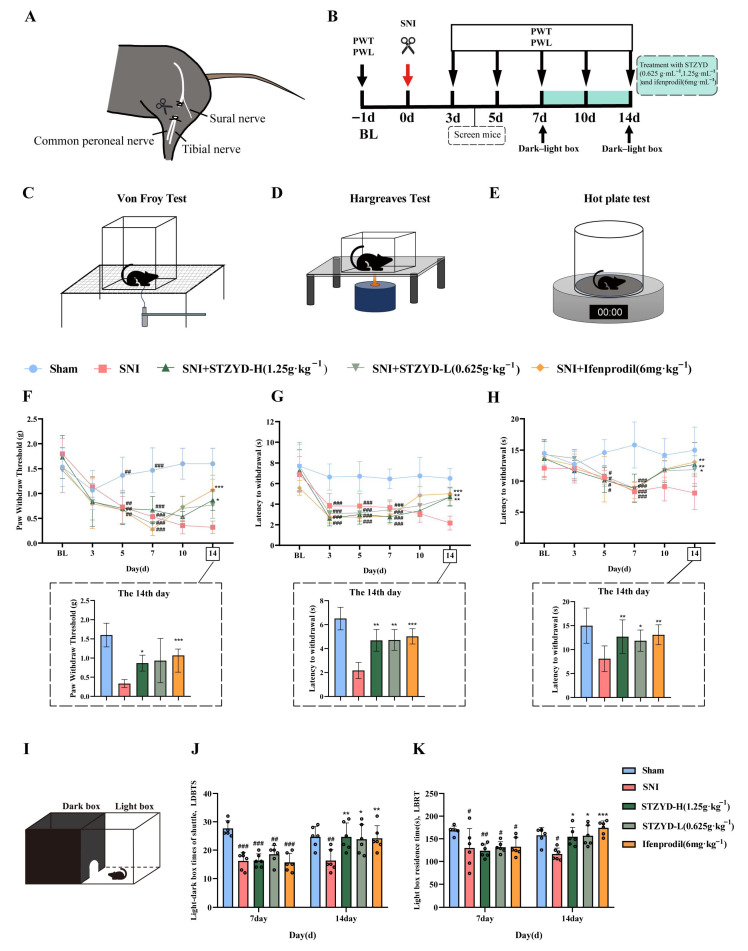
STZYD effects on pain behaviors in spared nerve injury (SNI) mice. (**A**) Schematic diagram of the spared nerve injury surgery, in which the tibial and common peroneal nerves were transected and ligated, while the sural nerve was preserved. (**B**) Timeline illustrating the schedule of the model establishment and behavioral testing. (**C**–**E**) These panels depict the schematic diagrams of the von Frey, Hargreaves, and hot-plate tests, respectively. (**F**) Mechanical pain thresholds of the plantar surface of the left hind paw measured over time in each group. The bar graph specifically represents the paw withdrawal threshold on day 14. (**G**) Paw withdrawal latency in response to thermal stimuli recorded using the Hargreaves test. The corresponding bar graph represents the paw withdrawal thermal latency on day 14. (**H**) Thermal paw withdrawal latency assessed in each group over time using the hot-plate test. The bar graph represents the latency on day 14. (**I**) Schematic diagram of the light–dark-box test. (**J**) The number of times mice in each group crossed the light–dark boxes in the light–dark-box experiments. (**K**) The residence time of mice in the light box in each group in the light–dark-box experiments. BL: Basal pain threshold of mice before modeling. One-way ANOVA was used to analyze all the behavioral data. All data are expressed as means ± SEMs (n = 6). # represents the comparison with the Sham group, and * represents the comparison with the SNI group. # *p* < 0.05, ## *p* < 0.01, and ### *p* < 0.001; * *p* < 0.05, ** *p* < 0.01, and *** *p* < 0.001.

As shown in Figure 2A,B, the spared nerve injury (SNI) model was successfully established in mice. On day 3 post-surgery, the model mice were screened for eligibility based on their behavioral performance. Behavioral assessments were conducted at baseline (BL) and on days 3, 5, 7, 10, and 14 after model induction. Schematic representations of the Von Frey, Hargreaves, and hot-plate test procedures are provided in Figure 2C–E. As shown in Figure 2F–H, one week following SNI surgery, both the paw withdrawal threshold (PWT) and paw withdrawal latency (PWL) were significantly decreased in the SNI group compared to those in the Sham group. After 7 days of treatment with STZYD or ifenprodil, both the paw withdrawal threshold and latency were significantly increased in the Von Frey, Hargreaves, and hot-plate tests, as compared to those of the SNI group (Figure 2F–H). These results indicate that treatment with high-dose STZYD (STZYD-H) and ifenprodil markedly attenuated mechanical and thermal hyperalgesia in the SNI mice.

In the light–dark-box test (Figure 2I), the number of transitions between compartments and the time spent in the light chamber were significantly reduced in the SNI group on day 7 post-surgery compared to the Sham group (Figure 2J). After 7 days of treatment with STZYD or ifenprodil, the number of transitions and the duration of stay in the light chamber were significantly increased in the treatment groups compared with the SNI group (Figure 2K).

### 2.3. STZYD Effects on Neurotransmitters

**Figure 3:**
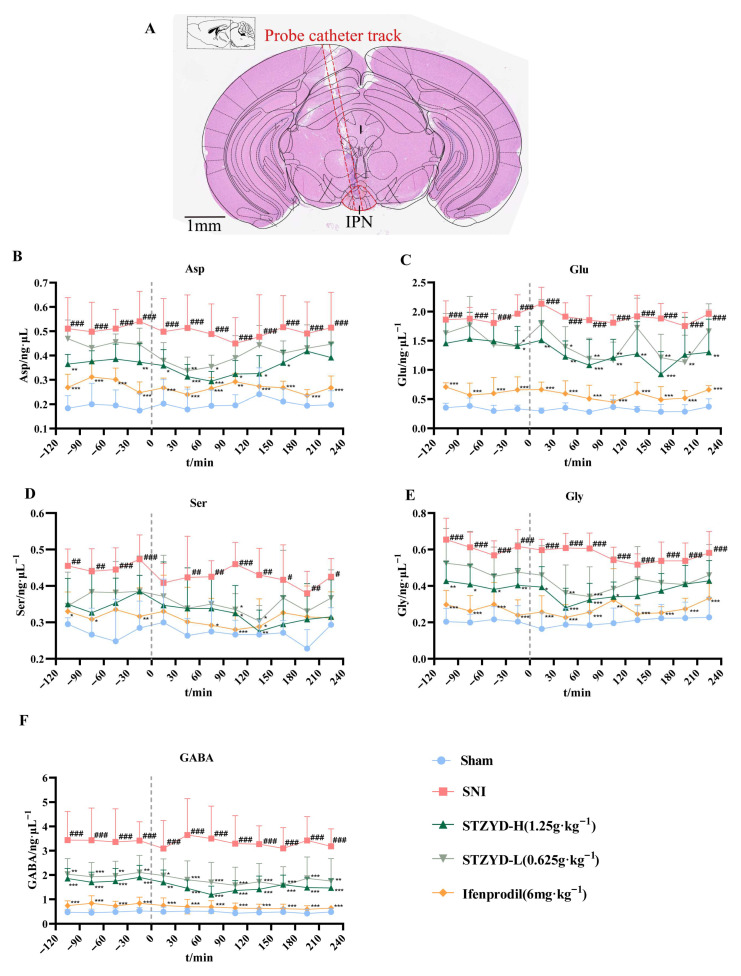
STZYD effects on amino acid neurotransmitters in interpeduncular nucleus (IPN). (**A**) Schematic diagram of the position of the microdialysis probe implanted in the IPN after HE staining. (**B**–**F**) STZYD effects on the Asp, Glu, D-Ser, Gly and GABA contents in the extracellular fluid of the IPN ($\bar{x}$ ± s; all data are expressed as means ± SEMs, n = 6). Two-way ANOVA was used to analyze the HPLC data. Compared with the Sham group, # *p* < 0.05, ## *p* < 0.01, and ### *p* < 0.001. Compared with the SNI group, * *p* < 0.05, ** *p* < 0.01, and *** *p* < 0.001.

In the microdialysis experiment, microdialysis guide cannulas were stereotaxically implanted into the IPNs of the mice (Figure 3A). Drug treatments were administered following probe insertion and a two-hour equilibration period. As illustrated in Figure 3B–F, compared with the Sham group, the SNI mice exhibited significantly elevated Asp, Glu, D-Ser, Gly, and GABA levels in their IPNs, indicating an imbalance in their excitatory and inhibitory neurotransmission following nerve injury. Both the STZYD-L and STZYD-H treatment groups had significantly reduced Asp levels from 0 to 180 min post-administration compared with the SNI group (Figure 3B). The ifenprodil group exhibited significant Asp level reductions over a longer period, from 0 to 240 min (Figure 3B). The STZYD-L and STZYD-H groups had significantly attenuated Glu levels from 0 to 240 min post-treatment (Figure 3C), and the reduction was even more pronounced in the ifenprodil group. The D-Ser levels were significantly reduced in both STZYD treatment groups from 90 to 150 min (Figure 3D). In the ifenprodil group, the decrease in the D-Ser levels occurred earlier and lasted longer, from 60 to 150 min. The STZYD-L and STZYD-H treatment groups exhibited significant Gly content reductions between 0 and 120 min (Figure 3E), while the ifenprodil group demonstrated more sustained decreases over 0 to 240 min. The GABA concentrations were significantly lowered in the STZYD-L and STZYD-H groups throughout the 0–240 min period (Figure 3F). The ifenprodil group showed similarly significant GABA level reductions across the same time window. These findings suggest that both the STZYD and ifenprodil treatments effectively modulated the neurotransmitter levels in the IPN regions of the SNI mice, potentially contributing to the alleviation of neuropathic pain and related behavioral symptoms.

### 2.4. STZYD Effects on Neuronal Activity

**Figure 4:**
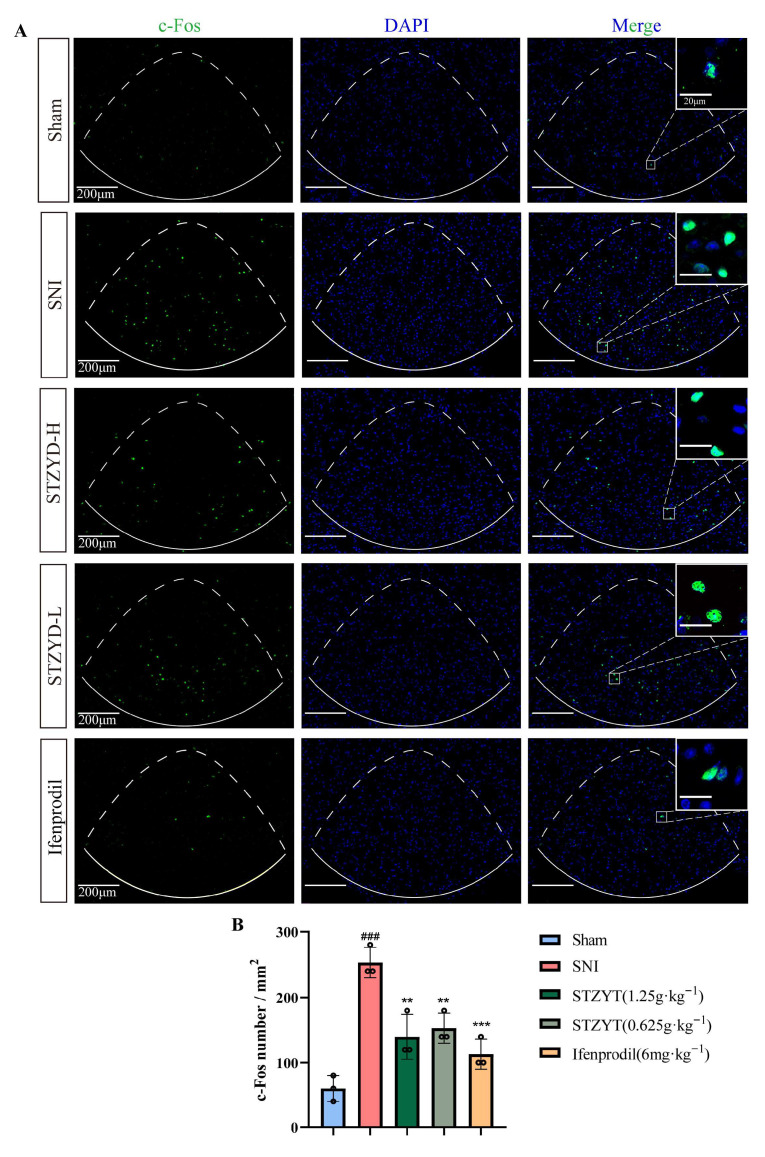
Regulation of c-Fos expression in the IPN via STZYD. (**A**) Representative immunofluorescence images showing c-Fos protein expression in the IPN across different experimental groups (scale bar: 200 μm; magnification: 10×; n = 3). (**B**) Quantification of c-Fos-positive neurons presented as number of labeled cells per mm^2^ of IPN tissue across treatment groups ($\bar{x}$ ± s; data are presented as means ± SEMs, n = 3). One-way ANOVA was used to analyze the number of cells in the immunofluorescence experiment. # represents the comparison with the Sham group, and * represents the comparison with the SNI group. ### *p* < 0.001, ** *p* < 0.01, and *** *p* < 0.001.

To evaluate the neuronal activation within the IPN, the c-Fos expression was examined using immunofluorescence staining (Figure 4A). c-Fos is an early gene product and is widely recognized as a marker of neuronal activation. As shown in Figure 4B, the c-Fos expression in the IPN was significantly upregulated in the SNI mice compared with that in the Sham-operated controls. Treatment with both L-STZYD and H-STZYD significantly reduced the c-Fos expression levels in the IPN. Similarly, the ifenprodil treatment also markedly decreased the c-Fos expression compared with that in the SNI group. These findings indicate that STZYD effectively suppressed neuronal activation in the IPN regions of the SNI mice, suggesting its potential mechanism in alleviating neuropathic pain symptoms.

**Figure 5:**
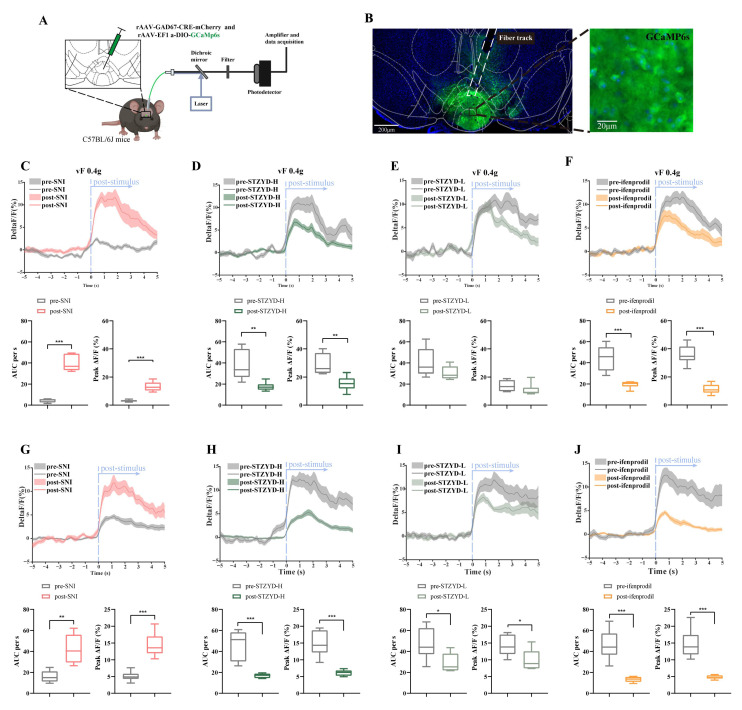
Integration of GCaMP expression and fiber photometry to measure the IPN GABAergic neuron activity during pain stimulation. (**A**) Schematic of real-time fiber photometry: rAAV-GAD67-CRE-mCherry and rAAV-EF1α-DIO-GCaMP6s were co-injected into the IPNs of C57 mice (300 nL in total). (**B**) Representative coronal section showing GCaMP6s expression in IPN neurons (green), with DAPI nuclear staining (blue). Scale bar: 200 μm; magnification: 2×. (**C**–**F**) Representative traces and quantification of GCaMP signals in response to von Frey stimulation (0.4 g), including area under the curve (AUC) and mean peak amplitude (ΔF/F_0_, n = 6). (**G**–**J**) Representative traces and quantification of GCaMP signals during the Hargreaves test, including AUC and mean peak amplitude (ΔF/F_0_, n = 6). Data are expressed as means ± SEMs. The AUC and mean peak amplitude were analyzed using the *t*-test. * *p* < 0.05, ** *p* < 0.01, and *** *p* < 0.001.

To investigate the in vivo activity of IPN GABAergic neurons in response to nociceptive stimuli, we employed the Cre-dependent gene delivery of the calcium indicator GCaMP6s into the IPNs of C57BL/6 mice using adeno-associated virus serotype 2/9 (AAV2/9) under the control of the GAD67 promoter (Figure 5A). The viral injection site within the brain region of the IPN is illustrated in Figure 5B. Fiber photometry was used to monitor calcium transients in response to Von Frey filament stimulation, enabling the real-time recording of neuronal activity. Three weeks after viral transduction, mice underwent SNI surgery. Calcium signals were recorded both prior to surgery and one week post-SNI to assess neuronal activity changes. Following postoperative recordings, mice received a one-week treatment with either high- or low-dose STZYD or ifenprodil, after which calcium imaging was conducted again. As shown in Figure 5C,G, mechanical stimulation using a 0.4 g Von Frey filament and thermal stimulation via radiant heat induced a robust increase in the GCaMP signals in the SNI model mice. Quantitative analysis of the recorded signals revealed a significant elevation in both the area under the curve (AUC) and mean peak amplitude compared to the preoperative baseline levels (Figure 5C,G), indicating the enhanced excitability of GAD67^+^ neurons within the IPN following peripheral nerve injury. Notably, treatment with high-dose STZYD or ifenprodil resulted in a marked reduction in GCaMP signals, as reflected by the significant decreases in both the AUC and mean peak amplitude (Figure 5D,F), suggesting that these interventions effectively attenuated injury-induced neuronal hyperactivity. In contrast, the low-dose STZYD group did not exhibit statistically significant GCaMP signal intensity, AUC, or peak amplitude changes, indicating the dose-dependent effect of the STZYD treatment. Interestingly, the low-dose STZYD group showed significant GCaMP signal reductions under thermal stimulation conditions (Figure 5I), with significant decreases in both the AUC and mean peak amplitude. Similarly, both the high-dose STZYD and ifenprodil treatment groups demonstrated the pronounced attenuation of heat-induced responses, as evidenced by the significantly reduced AUC and mean peak amplitude levels relative to the pre-treatment levels (Figure 5H,J).

### 2.5. Chemogenetic Modulation of GABAergic Neurons in IPN

**Figure 6:**
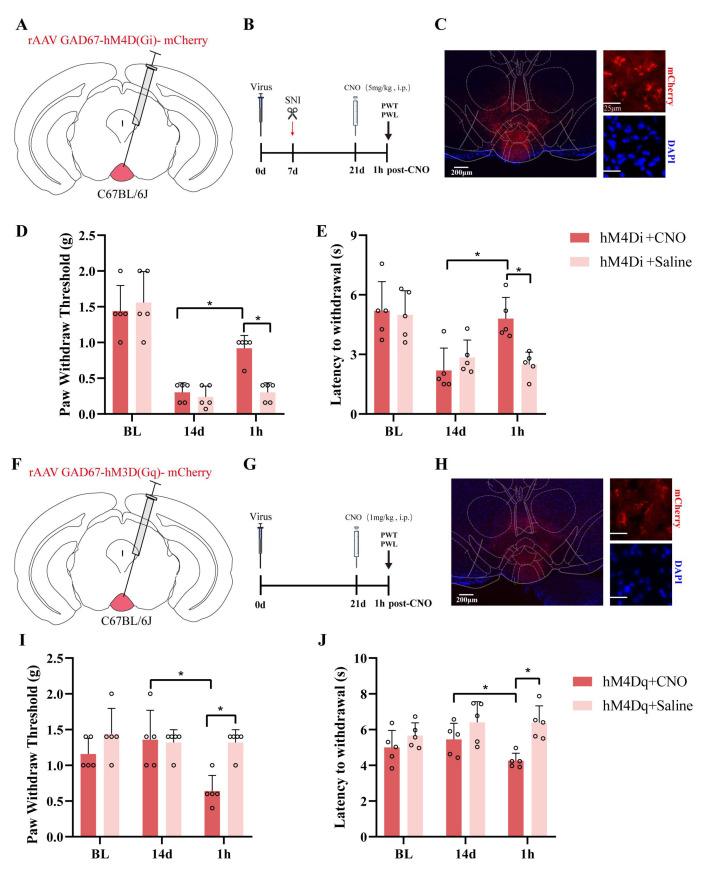
Effects of chemogenetic activation/inhibition of IPN GABAergic neurons on pain behavior. (**A**) Schematic representation of the viral (Gi) injection sites within the IPN (coronal section). (**B**) Experimental timeline illustrating the procedures. (**C**) Representative image showing hM3Di expression (red) in the IPN under the control of the GAD67 promoter; nuclei are counterstained with DAPI (blue). rAAV-GAD67-hM4D(Gi)-mCherry-WPREs (300 nL, 5.27 × 10^12^ vg/mL) were injected into the IPN. (**D**) Changes in mechanical pain thresholds at baseline, 14 days post-SNI, and following CNO administration in SNI and control mice. (**E**) Changes in thermal pain thresholds at baseline, 14 days post-SNI, and following CNO administration in SNI and control mice. (**F**) Schematic representation of the viral (Gq) injection sites within the IPN (coronal section). (**G**) Experimental timeline illustrating the procedures. (**H**) Schematic representation of the viral injection sites expressing hM3Dq (red) within the IPN (coronal section); nuclei are counterstained with DAPI (blue). rAAV-GAD67-hM4D(Gq)-mCherry-WPREs (300 nL, 6.04 × 10^12^ vg/mL) were injected into the IPN. (**I**) Changes in mechanical pain thresholds at baseline, 14 days post-injection, and following CNO administration in naïve and control mice. (**J**) Changes in thermal pain thresholds at baseline, 14 days post-injection, and following CNO administration in naïve and control mice. All data are expressed as means ± SEMs, n = 5. Compared with the hM4Di/hM4Dq+CNO group, * *p* < 0.05.

To achieve targeted chemogenetic manipulation, we employed viral vector-mediated gene delivery to selectively express designer receptors exclusively activated by designer drugs (DREADDs) in GABAergic neurons of the IPN. These engineered receptors—hM4D(Gi) for inhibition and hM3D(Gq) for activation—are selectively responsive to the synthetic ligand clozapine-N-oxide (CNO). We first assessed the effects of hM4D(Gi)-mediated inhibition on the nociceptive behavior in mice subjected to SNI (Figure 6A,B). Coronal schematics depict the precise IPN injection sites of AAV-DIO-hM4D(Gi)-mCherry under the control of the GAD67 promoter (Figure 6C). Immunofluorescence confirmed the robust, cell-type-specific expression of hM4D(Gi) (red fluorescence) in GAD67^+^ neurons. The systemic administration of CNO (3 mg/kg, i.p.) produced a significant elevation in both the PWT and PWL relative to the pre-treatment baselines (Figure 6D,E). By contrast, the saline-injected SNI controls showed no significant PWT or PWL changes (Figure 6D,E). We next evaluated the behavioral consequences of activating hM3D(Gq) in the IPN GABAergic neurons of uninjured mice (Figure 6F–H). Following CNO injection (3 mg/kg, i.p.), hM3D(Gq)-expressing mice exhibited significant PWT and PWL decreases compared to the baseline measurements, indicative of induced pain-like responses (Figure 6I,J), while no significant alterations were observed in the saline-treated controls (Figure 6I,J).

### 2.6. STZYD Effects on Glutamate Receptor Expression

**Figure 7:**
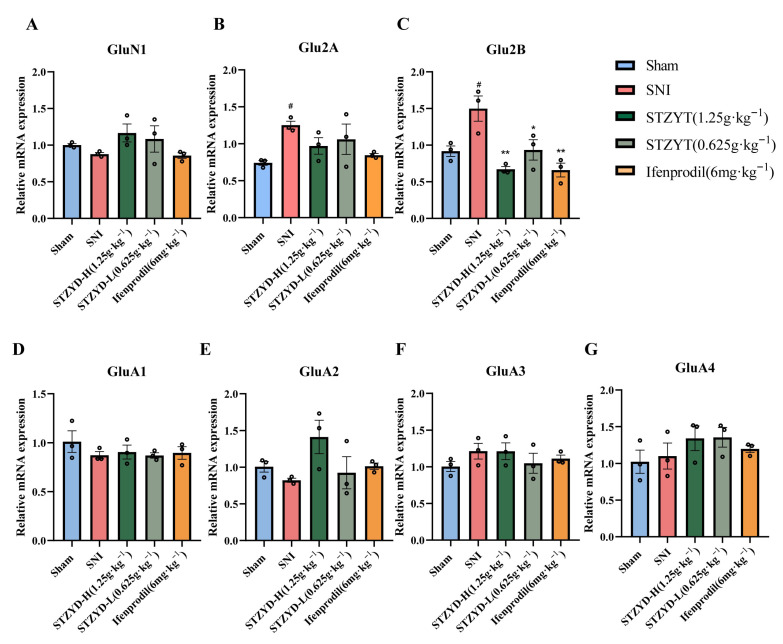
STZYD influence on glutamate receptor expression. (**A**–**G**) Relative mRNA levels of GluN1, GluN2A, GluN2B, and GluA1–GluA4 were quantified via RT-qPCR with GAPDH as the internal control. Data were analyzed by one-way ANOVA and are presented as means ± SEMs (n = 3). # represents the comparison with the Sham group, and * represents the comparison with the SNI group. # *p* < 0.05, * *p* < 0.05, and ** *p* < 0.01.

GAPDH was used as the housekeeping gene in the quantitative RT-qPCR experiment (Table 1). We compared the expressions of different glutamate receptor subunits in the IPN brain regions of the mice in each experimental group. As shown in Figure 7A–G, compared with the Sham group, the GluN2A and GluN2B expressions in the SNI group were significantly increased, while the mRNA GluN1, GluA1, GluA2, GluA3, and GluA4 contents did not change significantly. After treatment with STZYD and ifenprodil, the GluN2B expression was significantly reduced (Figure 7C). The above results indicate that STZYD can exert analgesic effects by inhibiting the mRNA expression levels of GluN2B receptors in SNI mice.

### 2.7. STZYD Effects on NMDAR-2B/CaMKII/CREB Signaling Pathway

**Figure 8:**
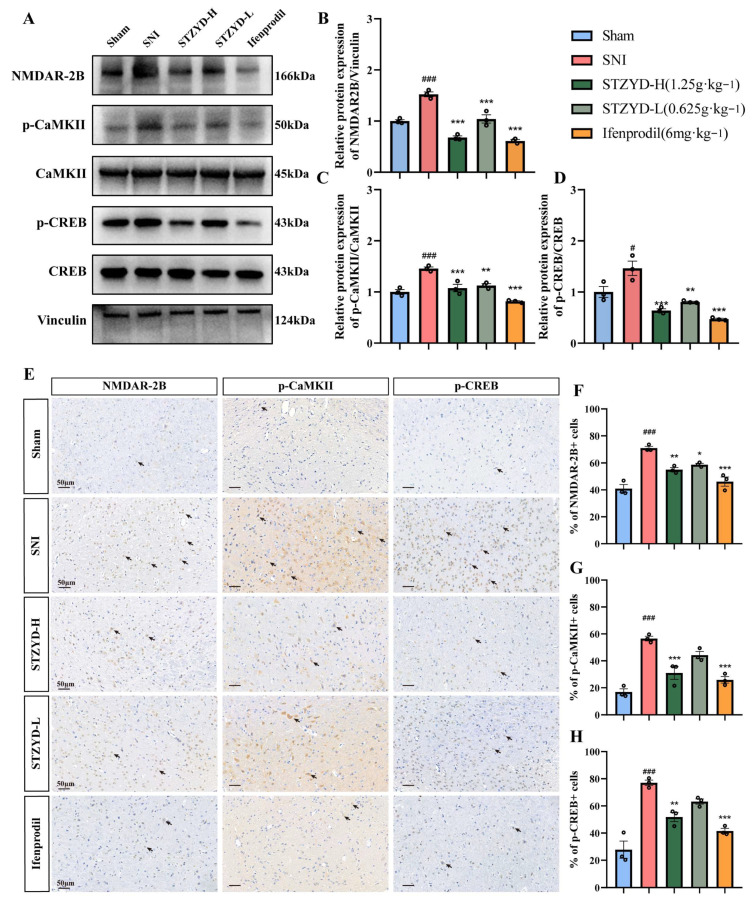
STZYD effects on the NMDAR-2B/CaMKII/CREB signaling pathway. (**A**) Western blotting was used to detect the expressions of NMDAR-2B, CaMKII, p-CaMKII, CREB, p-CREB, and Vinculin proteins in the IPN. (**B**) Relative protein expression levels of NMDAR-2B receptor. (**C**) Relative protein expression levels of p-CaMKII. (**D**) Relative protein expression levels of p-CREB. (**E**–**H**) Representative immunohistochemical images and statistical analysis of each group showing the positive areas of NMDAR-2B, p-CaMKII, and p-CREB in the IPN (scale bar: 50 μm; magnification 40×; n = 3). Black arrows indicate IPN neurons immunopositive for NMDAR-2B, p-CaMKII, or p-CREB. One-way ANOVA was used to analyze the protein expression levels and positive cell rates of NMDAR-2B, p-CaMKII, and p-CREB. All data are expressed as means ± SEMs, n = 3. Compared with the Sham group, # *p* < 0.05, and ### *p* < 0.001. Compared with the SNI group, * *p* < 0.05, ** *p* < 0.01, and *** *p* < 0.001.

Western blotting was performed with Vinculin as the internal control to assess STZYD’s effect on the NMDAR-2B/CaMKII/CREB signaling pathway (Figure 8A). According to the Western blot results, the NMDAR-2B, p-CaMKII, and p-CREB expression levels in the IPNs of the SNI group were significantly increased compared with those in the Sham group (Figure 8B). In contrast, the STZYD treatment significantly reduced the expressions of these proteins (Figure 8B–D). Furthermore, ifenprodil, an NMDAR-2B receptor antagonist, also significantly inhibited the phosphorylation of these proteins. Overall, these results indicate that STZYD inhibits the NMDAR-2B/CaMKII/CREB signaling pathway within the IPNs of SNI mice.

Immunohistochemistry confirmed the NMDAR-2B, p-CaMKII, and p-CREB expressions in the IPN (Figure 8E). The SNI group exhibited significant increases in the immunopositive expressions of these proteins compared with those of the Sham group (Figure 8F–H). Treatment with high-dose STZYD, low-dose STZYD, and ifenprodil significantly reduced the NMDAR-2B expression (Figure 8F), and the high-dose STZYD and ifenprodil groups also had significantly decreased p-CaMKII and p-CREB levels (Figure 8G,H). No significant changes in the p-CaMKII or p-CREB expression were observed in the low-dose STZYD group. These findings indicate that STZYD inhibits the NMDAR-2B/CaMKII/CREB signaling pathway.

### 2.8. Identification of STZYD Components

**Figure 9:**
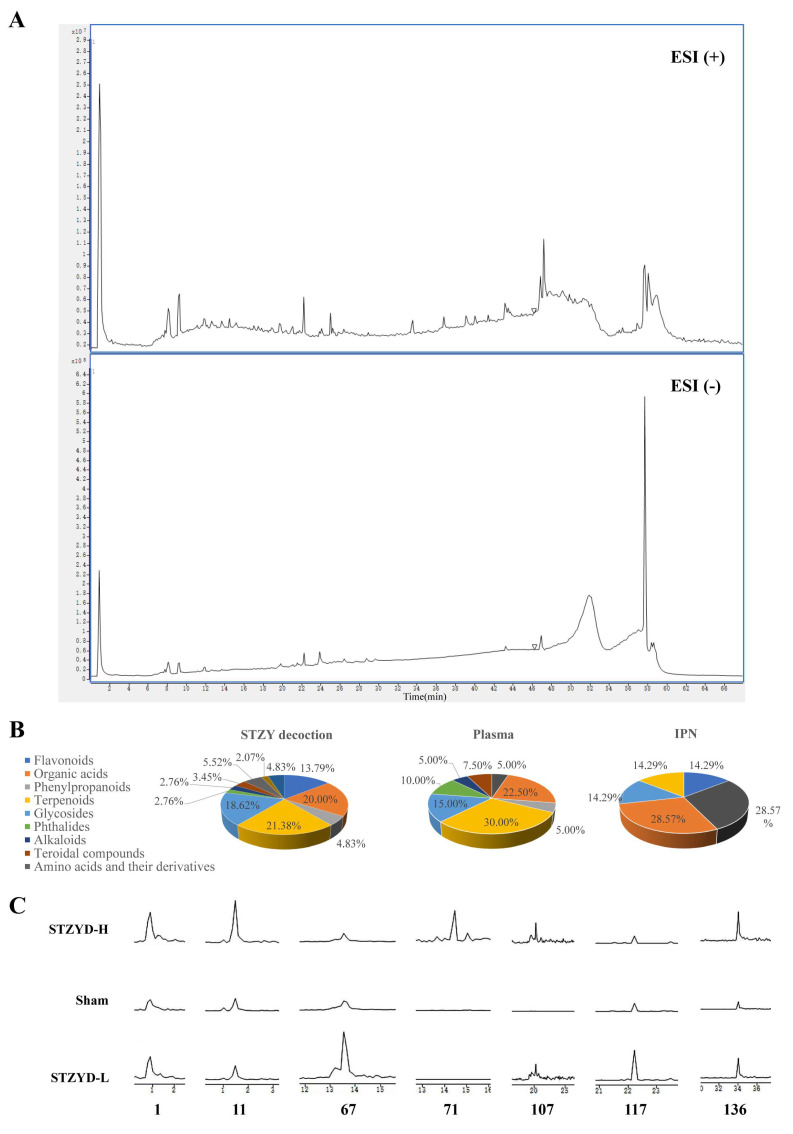
Identification of STZYD components. (**A**) Total ion chromatograms (TICs) of STZYD extracted with 50% methanol in both positive- and negative-ion modes, analyzed via HPLC-Q-TOF-MS/MS. (**B**) Pie chart showing the relative percentages of different chemical groups detected in STZYD, plasma, and INP samples via HPLC-Q-TOF-MS/MS. (**C**) EICs of components extracted from the IPNs of the STZYD-L, STZYD-H, and Sham groups. From left to right, the following compounds were sequentially detected: L-arginine, azelaic acid, formononetin, licoricesaponin K2, senkyunolide F, L-isoleucine, and lauric acid.

To identify the major active components, we conducted injection analyses on STZYD samples, serum samples from STZYD-treated mice, serum samples from control mice, and IPN tissues using the HPLC-Q-TOF-MS/MS technique. Extracted ion chromatograms (EICs) were obtained through comparative analysis. All detected components exhibited favorable separation and ionization efficiencies under both positive- and negative-ionization modes, as illustrated in Figure 9A. Detailed mass spectrometry data are provided in Table S1.

The identification of 145 compounds in STZYD was achieved by integrating multiple lines of evidence, including retention times, quasi-molecular ion peaks, tandem mass spectrometry fragmentation patterns, database matching using MassHunter PCDL Manager (Version B.08.00), and reference to the relevant literature. These compounds included 20 flavonoids (13.79%), 29 organic acids (20.00%), 7 phenylpropanoids (4.83%), 31 terpenoids (21.38%), 27 glycosides (18.62%), 4 phthalides (2.76%), four alkaloids (2.76%), five steroidal compounds (3.45%), 8 amino acids and their derivatives (5.52%), 3 nucleotides and related compounds (2.07%), and 7 other miscellaneous compounds (4.83%) (Figure 9B, Supplementary Table S1). As shown in Figure 9B, terpenoids and organic acids accounted for the largest proportions of the chemical composition, followed by glycosides and flavonoids, whereas alkaloids, phthalides, and steroidal compounds contributed relatively minor percentages. A total of 40 compounds were detected in the plasma, among which 7 were found to cross the blood–brain barrier and enter the IPN. The comparative pie charts (Figure 9B) also illustrate that terpenoids (30%) and organic acids (22.5%) were predominant in plasma, while amino acids and their derivatives (28.57%) and terpenoidal compounds (28.57%) were the major components detected in the IPNs, indicating the selective distribution pattern of the bioactive constituents. The ion current profiles of the extracted components from the IPN are shown in Figure 9C.

### 2.9. Molecular Docking Study

**Figure 10:**
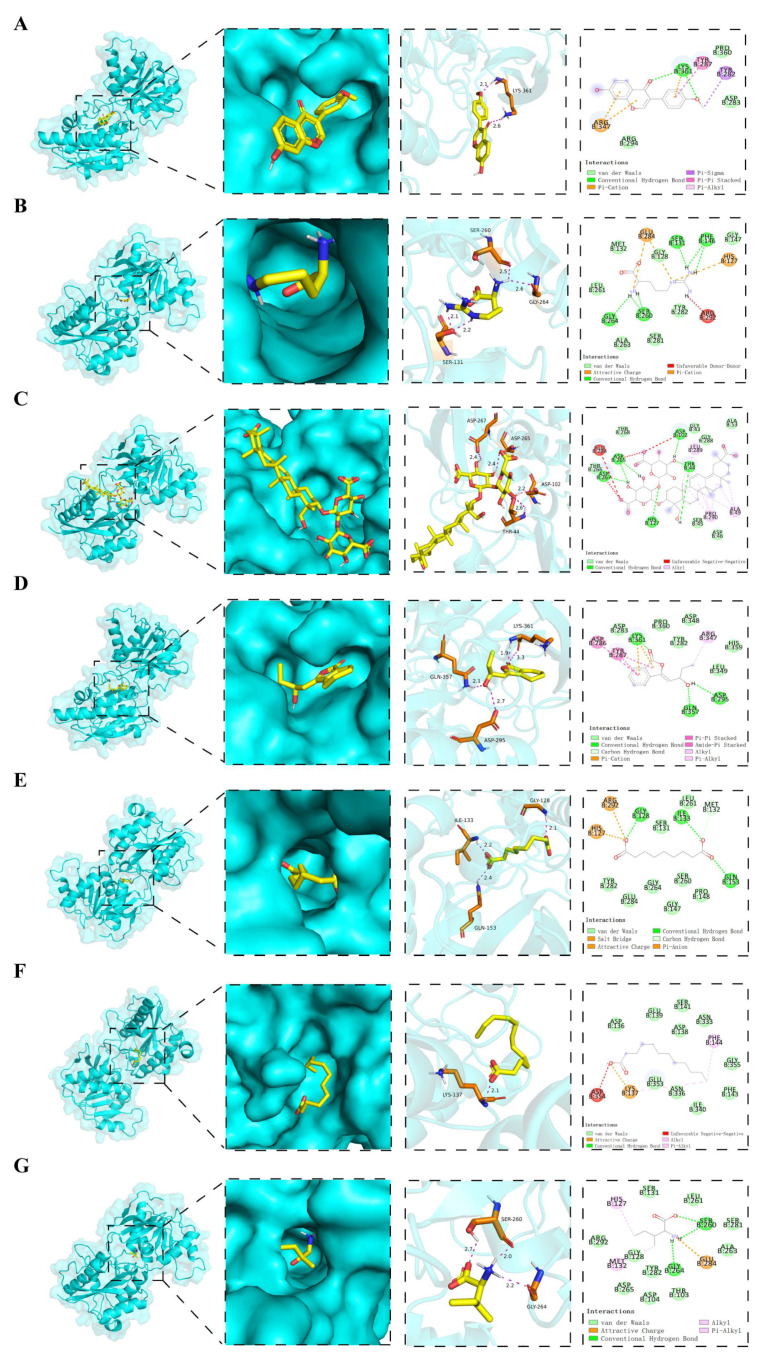
Molecular docking between NMDAR-2B and four pivotal ligands. The molecular docking results between NMDAR-2B and seven pivotal ligands, namely, (**A**) formononetin, (**B**) L-arginine, (**C**) licoricesaponin K2, (**D**) senkyunolide F, (**E**) azelaic acid, (**F**) lauric acid, and (**G**) L-isoleucine. Left: The small-molecule ligand (yellow) is embedded within the binding pocket of the NMDAR-2B subunit, with the receptor visualized as a cyan, solid surface. Middle: A stick model highlights key hydrogen bond interactions between the ligand (yellow) and critical residues; the receptor backbone is shown as a semi-transparent ribbon. Right: A two-dimensional schematic illustrates the ligand–protein interactions. Legend: green, solid lines—conventional hydrogen bonds; orange, dashed lines—salt bridges; red, dashed lines—attractive electrostatic interactions; light-green-filled circles—van der Waals forces; gray, dashed lines—carbon–hydrogen bonds; yellow, dashed lines—π–anion interactions.

Based on the HPLC-Q-TOF-MS/MS results, we conducted the molecular docking of the seven drug small molecules that entered the IPN region with the NMDAR-2B receptor. The docking scores of the compounds with the protein are shown in Table 2. Figure 10 depicts the images of the best docking of the receptor and ligand after visualization.

The results demonstrate that L-arginine, azelaic acid, formononetin, licoricesaponin K2, senkyunolide F, L-isoleucine, and lauric acid interacted with the NMDAR-2B receptor. As shown in Figure 10A, L-isoleucine formed three hydrogen bonds with the SER-260 and GLY-264 amino acid residues on NMDAR-2B, with bond lengths of 2.0 and 2.2 Å, respectively. Similarly, Figure 10B shows that azelaic acid formed three hydrogen bonds with GLY-128, ILE-133, and GLN-153, with bond lengths of 2.1, 2.2, and 2.4 Å, respectively. As shown in Figure 10C, lauric acid was predicted to establish one hydrogen bond with LYS-137, measuring 2.1 Å. As depicted in Figure 10D, senkyunolide F engaged the NMDAR-2B binding site through multiple interactions with critical residues, including GLN-357, LYS-361, and ASP-295. Hydrogen bonds within the range of 1.9–2.7 Å indicated strong binding affinities, while additional van der Waals and π–cation interactions further contributed to the stability of the ligand–receptor complex. According to the docking results, senkyunolide F showed a binding energy of <−5.0 kcal·mol^−1^ (Table 2), indicating a high-affinity interaction, which suggests that it may contribute to analgesic effects in the IPN brain region. As shown in Figure 10E, formononetin formed two hydrogen bonds with LYS-361 (2.1 and 2.6 Å) and established a π–cation interaction with ARG-347, as well as a π–π stacking interaction with TYR-287. Additionally, van der Waals contacts with PRO-360, ARG-294, and ASP-283 contributed to the binding stability. L-arginine formed multiple hydrogen bonds with SER-131, SER-260, GLY-264, and LEU-261 residues, with bond lengths ranging between 2.1 and 2.6 Å. Electrostatic interactions were observed with GLU-284 and HIS-127, while a π–cation interaction with TYR-282, along with hydrophobic contacts involving surrounding residues, further stabilized the binding conformation. Licoricesaponin K2 was predicted to bind within an open surface groove of the NMDAR-2B receptor, and its glycosidic moiety penetrated deeply into the binding cavity, forming stable hydrogen bonds with ASP-265, ASP-267, ASP-102, and THR-44, with bond lengths ranging from 2.2 to 2.6 Å, serving as key anchoring interactions. Furthermore, hydrophobic alkyl interactions with PRO-290 and ALA-49, together with surrounding van der Waals forces, enhanced the complex stability; however, despite electrostatic repulsion at GLU-284 and ASP-266, its impact on binding was minimal. The docking results showed that licoricesaponin K2 exhibited a stronger binding affinity (<−5.0 kcal·mol^−1^, Table 2), indicating excellent docking and high affinity. Therefore, we propose that licoricesaponin K2 exerts analgesic effects in the IPN brain region.

## 3. Discussion

NPP is one of the most intractable and debilitating forms of chronic pain, arising from disease or injury anywhere along the somatosensory pathway. From peripheral nociceptors to spinal dorsal horn neurons and from ascending tracts to cortical networks, maladaptive changes can initiate and perpetuate pain signaling at each level. In this context, central sensitization, driven by synaptic plasticity within the central nervous system (CNS), has emerged as the key mechanism by which transient peripheral insults are “memorized” as persistent pain. In this complex mechanism, network pharmacology provides a novel perspective. By integrating information at the molecular, cellular, and systems levels, network pharmacology facilitates the identification of key signaling pathways and therapeutic targets in NPP, thereby offering a multidimensional strategy for alleviating chronic pain. Accordingly, in this study, we first employed network pharmacology to predict the potential mechanisms by which STZYD exerts therapeutic effects on NPP, and we subsequently validated these predictions in animal experiments. Molecular, histological, and morphological analyses further demonstrated that NMDAR2B may serve as a critical membrane-associated target, suggesting that STZYD might modulate synaptic transmission in line with mechanisms involving NMDAR-mediated excitotoxicity and central sensitization. In addition, functional neuroscience experiments confirmed the role of GABAergic neurons within the IPN. Moreover, HPLC-Q-TOF-MS/MS analysis revealed that STZYD is capable of crossing the blood–brain barrier to reach the IPN and exhibits high affinity toward the NMDAR2B receptor.

Previous studies have demonstrated that STZYD exerts analgesic effects and modulates inflammation and immune responses primarily through the MAPK, PI3K/AKT, and NF-κB signaling pathways [19,20,23]; however, these investigations focused on spinal cord or DRG-level targets. Specifically, while studies such as [20,24] have shown that STZYD or its active ingredients modulate MAPK/TRPV1 signaling at the spinal cord or DRG level to alleviate neuropathic pain, our work provides the first evidence for a circuit-specific mechanism centered on IPN GABAergic microcircuits, offering a central mechanism of action that is independent of spinal pathways. Building on this finding, we employed network pharmacology to predict, and subsequently validate, the involvement of NMDAR-2B, thereby shifting the focus from traditional spinal MAPK/TRPV1 pathways to central pain modulation. In the present study, network pharmacology analysis revealed multiple enriched biological processes and signaling pathways, including cytokine regulation, lipopolysaccharide response, membrane raft organization, MAPK signaling, and AGE-RAGE signaling. Although these results suggest diverse mechanisms of action, many of the enriched terms converge both functionally and spatially on calcium-dependent synaptic signaling. GO enrichment analysis indicated the significant involvement of membrane- and synapse-related components (e.g., membrane rafts, postsynaptic density), receptor activity, and cytokine regulatory processes, all of which are positioned upstream or downstream of NMDAR-mediated calcium influx. Likewise, KEGG pathway analysis highlighted calcium signaling and glutamatergic synapse pathways, both of which explicitly include NR2B-containing NMDARs and CaMKII as key nodes. Notably, CaMKII activation can interface with MAPK and PKA/PKC cascades, providing multiple parallel routes for CREB phosphorylation. Thus, the enrichment of MAPK signaling is better interpreted as a collateral pathway converging on CREB rather than an independent mechanism. Taken together, the NMDAR-2B/CaMKII/CREB axis occupies a central hub position by integrating upstream synaptic receptor activation and coordinating downstream transcriptional programs.

Behavioral studies demonstrated that STZYD treatment significantly increased both the PWTs and PWLs in SNI mice, consistent with previous findings [20,25,26]. In the Von Frey test, low-dose STZYD did not yield a statistically significant effect. We posit that mechanical allodynia, compared with thermal pain, depends more on the sustained activation of spinal and supraspinal GluN2B-containing NMDA receptors. Partial target engagement at the low dose may suffice to attenuate thermal hypersensitivity but not to reverse mechanical allodynia, consistent with the significant increase in the mechanical withdrawal threshold observed only with the high dose. Moreover, thermal nociceptive pathways likely have a lower threshold for pharmacologic modulation and are thus more readily modulated by STZYD [27]. Anxiety-like behaviors were assessed using the light–dark-box test. Similarly, STZYD treatment showed a tendency to reduce anxiety-like behaviors [28], indicating its potential efficacy in alleviating both pain and anxiety. Given that STZYD attenuated both mechanical and thermal hypersensitivity in SNI mice, the increased time spent in, and transitions to, the light compartment in the light–dark box were likely secondary to analgesia rather than indicative of a primary anxiolytic effect. Demonstrating a pain-independent action will require testing STZYD in naïve, uninjured animals using validated anxiety paradigms (e.g., acute restraint or novelty-suppressed feeding). We will address this in future work by evaluating STZYD in assays specifically designed to isolate anxiety-related behaviors while minimizing confounding from analgesia.

Building upon these findings, we employed intracerebral microdialysis to examine neurotransmitter alterations in the IPN region. The IPN receives glutamatergic projections from the upstream mHb and has been implicated in pain and depressive behaviors [14,29]. Our results revealed significant elevations in the Asp, Glu, D-Ser, and Gly levels within the IPNs of SNI mice. The observed changes in the Asp and Glu levels may be associated with excitotoxicity following nerve injury [14,29], which aligns with our experimental hypotheses. Glycine or D-serine binding further stabilizes the receptor’s active conformation, facilitating ion channel opening [30]. Although studies have shown that prostaglandin E2 (PGE2) reduces glycine receptor responsiveness at the spinal level, particularly for receptors containing α3 subunits [31], its role in the midbrain may differ substantially. The concurrent elevation of excitatory and inhibitory neurotransmitters in the IPN after SNI reflects a maladaptive compensatory loop rather than a physiological balance. Peripheral nerve injury drives excessive glutamatergic input from the MHb, which, in turn, overactivates IPN GABAergic neurons and provokes disproportionate inhibitory release [11]. Concurrently, large amounts of GABA and Gly are released as compensatory inhibitory neurotransmitters, providing a negative feedback mechanism to counteract this hyperactivity; however, such compensation deviates from physiological homeostasis because its strength is directly coupled to excitatory input, thereby forming an abnormal cycle of “excess excitation → excessive inhibitory compensation” [12]. Our immunofluorescence, fiber photometry, and chemogenetic data indicate that STZYD interrupts this cycle by suppressing aberrant GABAergic overactivation and re-establishing synaptic equilibrium.

Studies have shown that c-Fos expression can promote the transcription of multiple pain-related genes, including c-Jun and dynorphin, whose upregulation contributes to the development and maintenance of neuropathic pain [32]. As a marker of neuronal activity, we detected elevated c-Fos protein levels in the IPNs of SNI mice, indicating IPN activation during pain processing. Emerging evidence [12,33,34,35] has demonstrated that anxiety-like behaviors induced by withdrawal increase the c-Fos expression in the IPN, enhancing the GABAergic neuronal activity and spontaneous firing frequency, but not glutamatergic neurons. Similarly, acute restraint stress increases the c-Fos expression in the IPN [36]. Given that anxiety and stress often accompany pain responses, it is plausible that the IPN plays a comparable role in pain modulation, particularly by regulating emotional responses that influence pain perception.

To further investigate GABAergic neurons, we recorded robust GCaMP signal fluctuations in the IPN of freely moving SNI mice upon subthreshold mechanical stimulation (0.4 g). Notably, the high-dose STZYD treatment effectively attenuated this GCaMP signal increase, consistent with previous reports of its analgesic effects in neuropathic pain models [20]. In this study, we achieved enhanced analgesia using ifenprodil, an NMDA receptor antagonist that selectively inhibits receptors containing the NR2B subunit. Ifenprodil is widely used in the study of NMDA receptor subtypes and has demonstrated potent analgesic effects in both acute and chronic pain models with minimal side effects [37,38,39,40]. These findings suggest that STZYD may exert its analgesic effects by modulating GABAergic neurons via the NMDAR-2B receptor.

Furthermore, we successfully utilized chemogenetic technology to achieve the targeted manipulation of GABAergic neurons in the IPN, systematically exploring the functional role of this brain region in pain modulation. By selectively expressing the inhibitory receptor hM4D(Gi) and the excitatory receptor hM3D(Gq), in combination with CNO administration, we revealed the bidirectional regulation of nociceptive behavior mediated by IPN GABAergic neurons in both chronic neuropathic pain and pain induction models. In the SNI model, activation of the inhibitory DREADD receptor hM4D(Gi) significantly increased the PWT and PWL, suggesting that the inhibition of GABAergic neurons in the IPN can alleviate pain-related behaviors. These findings support the inhibitory role of the IPN in chronic pain modulation and are consistent with previous studies showing the involvement of the IPN in emotional and reward circuit regulation. Additionally, our use of a GAD67 promoter-driven viral vector strategy ensured high expression specificity, which enhanced the precise dissection of the GABAergic neuronal function in this brain region. Interestingly, activation of the excitatory DREADD receptor hM3D(Gq) in uninjured mice resulted in significant reductions in both the PWT and PWL, indicating that the excessive activation of GABAergic neurons alone can induce pain-like responses, which suggests that under normal conditions, IPN GABAergic neurons may play a potential pronociceptive role, and that their overactivity could amplify pain signals under pathological conditions. These findings complement the analgesic effects observed in the hM4D(Gi) group, supporting the plasticity and bidirectional regulatory function of the IPN in pain perception. Although the chemogenetic data demonstrate bidirectional pain control via IPN GABAergic neurons, the initial n = 5 per group limits the statistical power; an independent replication (total n ≈ 10–11) and larger follow-up studies are required to confirm the reproducibility.

As a “molecular switch” of central sensitization, the NMDAR integrates peripheral nociceptive signals, central plasticity, and emotional regulation through a “subtype-spatiotemporal-specific” mechanism. Research shows that NMDAR upregulation and hyperactivation occur across diverse pain models, including spinal cord injury and inflammatory pain [41,42,43,44,45,46,47,48,49,50]. Notably, the NR2A and NR2B subunits are implicated in nociceptive transmission within key pain-processing regions: the caudal subnucleus of the spinal trigeminal nucleus, dorsal root ganglia (DRG), spinal dorsal horn, prefrontal cortex, and amygdala [44,45,46,47,48,49,50]. The GRIN2B (encoding GluN2B) rs1806201 variant has been associated with susceptibility to chronic postsurgical pain, suggesting that NMDAR subtype expression levels may serve as biomarkers for pain classification and therapeutic response prediction [51]. Our study further demonstrated that the mRNA and protein levels of the NMDA2B subunit, but not of other subtypes, were significantly elevated in the IPNs of the SNI mice. Notably, both the high-dose STZTD treatment and ifenprodil administration effectively downregulated this enhanced NMDA2B expression. These findings suggest that the excessive release of amino acid neurotransmitters (e.g., glutamate) may overactivate excitatory receptors, such as the NMDAR, leading to pathological calcium influx in neurons. This cascade exacerbates neural damage and perpetuates maladaptive pain signaling [52]. Rather than relying on a single “key molecule”, the in vivo efficacy of STZYD likely stems from additive or synergistic GluN2B occupancy by licoricesaponin K2 and senkyunolide F, coupled with allosteric modulation via lower-affinity ligands such as azelaic acid and L-arginine. This multicomponent, multi-site binding mode may explain why the high-dose decoction, achieving the delivery of a combined compound mixture at supra-threshold brain levels, attains the complete suppression of the GluN2B/CaMKII/CREB axis, an effect rarely achieved with individual NMDAR blockers.

Electrochemical signaling plays a pivotal role in the central nervous system. As ligand-gated, voltage-dependent ion channels, hyperactivated NMDARs mediate pathological Ca^2+^ influx. When an action potential reaches the axonal terminal, the Ca^2+^/CaM complex binds to the autoinhibitory domain of CaMKII, relieving its autoinhibition and triggering autophosphorylation-mediated activation [53]. Activated CaMKII phosphorylates CREB at Ser133 [39], enabling its binding to cAMP response elements (CREs) and the subsequent recruitment of RNA polymerase II to form transcriptional complexes that regulate downstream gene expression [54]. CREB activation promotes the transcription of multiple pain-related genes, including c-Fos, c-Jun, NK-1, COX-2, and BDNF [39,54]. The upregulation of these genes contributes to central sensitization—the neurobiological foundation underlying the transition from acute injury to chronic pain.

Our results demonstrate that STZYD exerts potent, dose-dependent antinociception in the SNI model by silencing the peripheral NMDAR-2B/CaMKII/CREB axis. The rapid upregulation of NMDAR-2B and the concomitant phosphorylation of CaMKII and CREB observed in the IPN following nerve injury mirror the cascade reported in diabetic and chemotherapy-induced neuropathy models, where this pathway drives mechanical hypersensitivity [48,55,56,57]. STZYD reversed these molecular changes at both the protein and phosphorylation levels, indicating that it disrupts the entire receptor kinase–transcription factor signaling axis rather than acting at a single node. The high-dose regimen produced a complete suppression of p-CREB, a finding rarely achieved by classic NMDAR antagonists that merely attenuate channel gating. This downstream shut-off suggests that STZYD may prevent the transcription of pronociceptive genes (c-Fos, BDNF, COX-2), a mechanism recently highlighted in chronic pain epigenetic studies [58]. In contrast, the low-dose group inhibited NMDAR-2B expression without altering p-CaMKII/p-CREB, implying that additional anti-inflammatory or antioxidant constituents of STZYD become indispensable for full pathway blockade at higher concentrations. Such multitarget synergy is reminiscent of kaempferol, which simultaneously modulates Nrf2 and NF-κB to dampen neuropathic pain [58,59]. In contrast to conventional NMDAR antagonists, which predominantly target the central nervous system and can induce cognitive or motor side effects, STZYD, as a traditional orally administered Chinese medicine, demonstrates a more favorable safety profile [60]. Collectively, whereas GABA_B agonists (e.g., baclofen) only partially dampen CREB phosphorylation, STZYD achieves the comprehensive shut-down of the NMDAR-2B/CaMKII/CREB signaling chain.

Using HPLC-Q-TOF-MS/MS, we identified seven constituents that crossed the blood–brain barrier (BBB) and accumulated in the IPN: L-arginine, azelaic acid, formononetin, licoricesaponin K2, senkyunolide F, L-isoleucine, and lauric acid. Given that the NMDAR-2B/CaMKII/CREB axis is a key mediator of central sensitization, we focused on investigating whether these BBB-penetrant compounds modulate NMDAR-2B to contribute to STZYD’s analgesic effects. Consistent with this hypothesis, ifenprodil, a selective NMDAR-2B antagonist, replicated STZYD’s analgesic efficacy, validating NMDAR-2B as a viable target for pain modulation. The molecular docking of the seven compounds to NMDAR-2B revealed that licoricesaponin K2 and senkyunolide F exhibited the strongest binding affinities, suggesting stable receptor–ligand engagement. Both compounds formed key hydrogen bonds and hydrophobic and π–π stacking interactions, stabilizing NMDAR-2B in an inactive conformation and preventing its activation at the glutamate- and glycine-binding sites. This high-affinity binding impedes NMDAR-2B channel opening, thereby reducing pathological Ca^2+^ influx. Licoricesaponin K2, with a binding energy of −8.2 kJ/mol, demonstrated stable binding via hydrogen bonds and hydrophobic interactions, with its glycosidic moiety deeply penetrating the receptor’s binding cavity, enhancing its therapeutic potential for pain. Similarly, senkyunolide F, with a binding energy of −7.8 kJ/mol, formed multiple interactions, including hydrogen bonds, van der Waals forces, and π–cation interactions, supporting its potential as a potent NMDAR-2B modulator for pain management. The diverse nature of its binding interactions suggests that senkyunolide F could exert a more stable and effective influence on receptor activity, making it a promising candidate for further preclinical evaluation. These interactions are consistent with the mechanism proposed for ifenprodil and related NMDAR-2B selective antagonists, which prevent glutamate/glycine co-agonist binding, and thus block Ca^2+^ influx and downstream CaMKII/CREB signaling [60,61,62]. Integrating in vivo data, including the complete suppression of the NMDAR-2B/CaMKII/CREB axis and dose-dependent analgesia, we hypothesize that licoricesaponin K2 is the primary active constituent, with the other compounds acting as allosteric modulators to enhance the overall inhibitory potency. This multicomponent, multitarget binding mode underpins the superior efficacy of STZYD compared with single-target NMDAR-2B blockers.

We acknowledge that the 12-herb STZYD composition was not analytically standardized across batches and that analgesic end-points were assessed only within 24 h after the last daily dose. Consequently, the metabolite variability, long-term PK, and effect duration remain undetermined and will be addressed in future marker-controlled extract studies.

We posit that STZYD should serve as an adjuvant to standard NMDA antagonists or GABA mimetics, rather than as a monotherapy, to reduce their required doses. High-dose STZYD completely suppressed the GluN2B/CaMKII/CREB axis with broader reach than ifenprodil. We hypothesize that when combined, STZYD and traditional NMDA antagonists may achieve the same level of inhibition at lower doses, benefiting from both direct blockade and allosteric synergy. This could limit adverse CNS events, such as cognitive impairment, which are common with high-dose NMDA antagonists. Unlike the existing GluN2B-selective drugs that target nociception only, STZYD simultaneously alleviates pain and comorbid anxiety, offering a dual analgesic–anxiolytic profile aligned with multidimensional pain management. Bioactive constituents (licoricesaponin K2, senkyunolide F) are small, BBB-permeant GluN2B ligands that can be engineered into targeted probes for optimized co-formulations. Regarding the pharmacokinetics of STZYD, while 40 compounds entered murine plasma and 7 reached the IPN, their human pharmacokinetics remain uncharacterized. The concentration of a single component in the body is insufficient to produce a significant effect. However, the seven components achieve the transformation from a low-dose combination to efficient pathway regulation through additive or synergistic effects, which represents the core advantage of the multi-mechanistic properties of compound traditional Chinese medicine. We recognize that future studies should focus on the human oral bioavailability, steady-state brain exposure, major metabolites, drug–drug interaction potential, and chronic toxicology before clinical use. Furthermore, we will include a more detailed discussion on the possible synergistic effects of these compounds in STZYD, as polypharmacology may contribute significantly to its therapeutic efficacy.

## 4. Materials and Methods

### 4.1. Animals

Male C57BL/6J mice (19–22 g) were obtained from Beijing Huafukang Biotechnology Co., Ltd. (Beijing, China), and maintained in the lab animal center of the Institute of Basic Theory for Chinese Medicine, China Academy of Chinese Medical Sciences. Animals were kept at 20–24 °C and 40–50% relative humidity under a 12 h light–dark cycle with ad libitum access to food and water. All animal procedures were approved by the Animal Care and Use Committee of the Experimental Research Center of the China Academy of Chinese Medical Sciences (Approval No. ERCCACMS21-2405-01) and were performed in strict accordance with the institutional guidelines for laboratory animal welfare.

### 4.2. Network Pharmacology Analysis

The active ingredients of STZYD were identified through the Traditional Chinese Medicine Systems Pharmacology Database and Analysis Platform (TCMSP, https://old.tcmsp-e.com/tcmsp.php, accessed on 24 September 2024). The chemical constituents of the 12 herbs in STZYD were retrieved, and potential bioactive compounds were selected based on two key pharmacokinetic parameters: their oral bioavailability (OB ≥ 30%) and drug-likeness (DL ≥ 0.18). Target proteins for these active compounds were identified via TCMSP and further cross-referenced with the UniProt database (http://www.uniprot.org/, accessed on 22 August 2024) to standardize the gene names and ensure accuracy.

Disease targets associated with NPP were gathered from the GeneCards database (https://www.genecards.org/, accessed on 24 August 2024), the Human Mendelian Inheritance Database (OMIM, http://www.omim.org, accessed on 24 August 2024), and the DrugBank database (https://www.drugbank.ca, accessed on 24 August 2024). Redundant targets were removed, leaving only unique disease-related targets for further analysis.

The intersection of the STZYD active compound targets and NPP-related disease targets was determined using jvenn (https://jvenn.toulouse.inra.fr/app/index.html, accessed on 24 August 2024), resulting in the identification of overlapping targets between the drug and disease.

To construct a “traditional Chinese medicine–active-ingredient–target–disease “ network, the identified active components and NPP-related targets were imported into Cytoscape software. Additionally, potential target proteins for STZYD in the treatment of NPP were predicted using the STRING database (https://string-db.org/, accessed on 26 August 2024), with the species set to Homo sapiens and a confidence threshold of 0.400. The resulting protein–protein interaction (PPI) network was visualized, and the degree centrality (node connectivity) of each protein was calculated using the “Analyze Network” plugin in Cytoscape to identify core targets.

The intersecting drug–disease targets were further analyzed using the Metascape platform (https://metascape.org, accessed on 26 August 2024). Gene Ontology (GO) enrichment analysis was performed, focusing on biological processes (BPs), molecular functions (MFs), and cellular components (CCs), to elucidate the role of STZYD’s target proteins in treating NPP. Kyoto Encyclopedia of Genes and Genomes (KEGG) pathway enrichment analysis was conducted to identify key signaling pathways involved in the treatment of NPP via STZYD. The most significant gene functions and pathways were visualized through bar and bubble charts.

### 4.3. Spared Nerve Injury Surgery

The spared nerve injury (SNI) surgery strictly followed the requirements of the International Association for the Study of Pain for conducting pain experiments on awake animals. Male C57BL/6J mice were first induced with 4% isoflurane and then maintained at 1.1% isoflurane (0.15 L·min^−1^) via a gas anesthesia machine. All surgical instruments were sterilized via high temperature. After the mice were completely anesthetized, the hair on the left knee joints of the mice was shaved off, and a 0.5–1 cm incision was made with scissors at the muscle bulge to expose the sciatic nerve bundle comprised the tibial nerve, common peroneal nerve, and sural nerve. The tibial and common peroneal nerves were ligated with surgical suture and then cut 1–2 mm distally, leaving only the sural nerve. In the Sham group, the mice underwent a similar procedure but without nerve injury. After the surgery, gentamicin (0.01 mL·10 g^−1^) was injected into the mice to prevent infection, and then the mice were returned to the cage. Seven days after surgery, mice were randomly assigned to one of five groups (n = 6 per group): the Sham, SNI, SNI + low-dose STZYD (0.625 g·mL^−1^), SNI + high-dose STZYD (1.25 g·mL^−1^, expressed as crude drug), or SNI + ifenprodil (6 mg·kg^−1^, i.p.) groups. Treatments were administered daily for seven consecutive days; the Sham and SNI groups received equivalent volumes of normal saline.

### 4.4. STZYD Preparation

STZYD was administered to mice by oral gavage once daily for 7 days at 0.625 g·mL^−1^ (0.2 mL·10^−1^ g body weight). The dosing regimen was based on the Chinese Pharmacopoeia recommendation of 15 g crude drug/60 kg adult (0.25 g·kg^−1^). According to the FDA interspecies body–surface area conversion (mouse factor: 12.3), the equivalent mouse dose was 0.625 g·kg^−1^ (low-dose group), and the high-dose group received 1.25 g·kg^−1^ (2× clinical exposure). STZYD consists of the following twelve traditional Chinese medicinal herbs, all of which were purchased from Beijing Tongrentang (Beijing, China) to ensure consistent quality for the subsequent experimental procedures: *Gentiana straminea* Maxim. (Qinjiao, QJ), 3 g; *Ligusticum chuanxiong* Hort. (Chuanxiong, CX), 6 g; *Prunus davidiana* (Carr.) Franch. (Taoren, TR), 9 g; *Carthamus tinctorius* L. (Honghua, HH), 9 g; *Glycyrrhiza uralensis* Fisch. (Gancao, GC), 6 g; *Notopterygium incisum* Ting ex H. T. Chang (Qianghuo, QH), 3 g; *Commiphora molmol* Engl. (Moyao, MY), 6 g; *Angelica sinensis* (Oliv.) Diels (Danggui, DG), 9 g; *Trogopterus xanthipes* Milne-Edwards (Wulingzhi, WLZ), 6 g; *Cyperus rotundus* L. (Xiangfu, XF), 3 g; *Achyranthes bidentata* Bl. (Niuxi, NX), 9 g; *Pheretima aspergillum* (E. Perrier) (Dilong, DL), 6 g. First, 135 g of QJ, QH, and XF, 270 g of CX, GC, MY, WLZ, and DL, and 405 g of TR, HH, DG, and NX were taken. Then, they were added to 10 times the volume of distilled water (31 L), soaked for 1 h, and heated in a sealed container, and after boiling, the extract was refluxed for 1 h [18,19,20]. For the second time, 8 times the volume of distilled water (24.84 L) was added. After the pot boiled, it was sealed and refluxed for 1 h. The two extracts were combined, filtered, and concentrated in a low-temperature cycle. The solution was then made up to 5.2 L. An amount of 1.5 L of concentrate was reserved for the low-dose decoction (0.625 g·mL^−1^), and the remainder was concentrated to obtain the high-dose decoction (1.25 g·mL^−1^). For chemical profiling, 5 g of each herb was finely powdered and extracted with 70% methanol via sonication for 30 min. After centrifugation at 13,000 rpm for 15 min, the supernatant was collected and diluted appropriately with 50% methanol, and a 50 μL aliquot was analyzed via HPLC-Q-TOF-MS/MS.

### 4.5. Preparation of Mouse Serum and Brain Tissue Samples

The SNI mice in the high- and low-dose STZYD groups were administered the STZYD via gavage (0.2 mL·10 g^−1^), while the Sham group was given the same volume of normal saline via gavage. The doses were continuously administered for 3 d to ensure that the marker components reached steady-state plasma levels and to avoid potential metabolite degradation or nonspecific tissue interference due to long-term administration [63]. On the last day, blood was collected from the orbital vein, and the brain was removed 2 h after the administration. The blood samples were left to stand at room temperature for 2 h, the samples were then centrifuged at 3500 rpm and 4 °C for 15 min, and the supernatant was taken. The brain tissue was sliced using a mouse brain mold, and the IPN brain region was extracted. The IPN tissue was precisely weighed and homogenized with 2 times the volume of normal saline. The aforementioned prepared samples were subjected to HPLC-Q-TOF-MS/MS analysis.

For in vivo pharmacodynamic studies, all tissue samples were obtained after 7 days to ensure that the therapeutic effect of the drug had stabilized. After the behavioral test, three mice from each group were selected for the Western blotting and RT-qPCR experiments to detect proteins and mRNA, and the remaining three mice were used for immunohistochemical staining. After the microdialysis experiment, the brain tissues of the mice were taken and fixed with 4% paraformaldehyde for HE staining to assess the probe implantation site. The c-Fos protein detection was carried out after the behavioral test, and the brain tissue was removed via cardiac perfusion and fixed.

### 4.6. Behavioral Tests

All mice were acclimated to the testing room environment for 3 h prior to behavioral testing. To ensure consistency, the same experimenter conducted all the procedures.

#### 4.6.1. Von Frey Hairs Test

The mice were placed in transparent plastic boxes (10 cm × 5.8 cm × 10 cm) on the wire mesh for at least 30 min to familiarize themselves with the experimental environment. When Von Frey filaments were applied vertically to the metacarpal and calcaneal junction of the left hind paw, painful reactions such as brisk paw withdrawal, flinching, licking, or shaking were considered positive. The minimum fiber gram value when the mouse positive reaction reached three out of five stimulation times was identified as the mechanical pain threshold response value, and the interval of each measurement was at least 1 min. If there was no positive reaction, a higher gram weight fiber was used for stimulation until a positive reaction occurred. The positive values were denoted as the paw withdrawal threshold (PWT).

#### 4.6.2. Hargreaves Test

Thermal sensitivity was assessed using the Hargreaves and hot-plate tests. During testing, an infrared radiant heat source (55 W) (Ugo Basile 21036, Gemonio, VA, Italy) was applied to the central plantar surface of the left hind paw. The latency to paw withdrawal or licking in response to thermal stimulation was recorded as the paw withdrawal latency (PWL). Thermal laser stimulation was limited to a maximum duration of 20 s to prevent tissue damage. Each animal was tested at 10 min intervals for at least three trials, and the average value of the three measurements was calculated as the final PWL for each mouse.

#### 4.6.3. Hot-Plate Test

The hot-plate test was used to assess the thermal nociception by measuring the PWLs in the mice. Animals were individually confined in transparent acrylic cylinders on a heated surface maintained at 55.0 ± 0.1 °C. The PWL was defined as the time elapsed until the manifestation of nociceptive behaviors, including hind paw licking, shaking, or lifting. An automatic 30 s cutoff was implemented to prevent tissue injury, with the manual removal of non-responsive mice at this threshold. Tests were conducted at 10 min intervals to allow for recovery between trials.

#### 4.6.4. Light–Dark-Box Test

The light–dark-box test was conducted using a 21 cm × 21 cm × 45 cm box. The left side of the box was black and the right side was a white chamber with illumination. The mice were allowed to move freely between the two compartments for 5 min. A motion-tracking system recorded the light-box residence time (LBRT) and the number of transitions between the light and dark compartments. Data analysis was performed using Shanghai Jiliang software (version 2021, Shanghai, China).

### 4.7. Stereotaxic Surgery

Mice were deeply anesthetized with intraperitoneal tribromoethanol (0.2 mL·10 g^−1^, i.p.). After shaving the scalps, animals were positioned in a stereotaxic frame (RWD Life Science Co., Ltd., Shenzhen, China) on a thermostatically controlled heating pad (KEL-2000, Nanjing, China) to maintain their core temperatures. After leveling the skull, we targeted the interpeduncular nucleus (coordinates: AP: −3.52 mm; ML: −1.25 mm; DV: −4.53 mm; 16° right angle), drilled a craniotomy with a skull drill (RWD Life Science Co., Ltd., Shenzhen, China), and secured the microdialysis probe guide cannula to the skull using screws and dental cement. Postoperatively, each mouse received gentamicin (0.01 mL per 10 g, s.c.) and was housed individually.

In the fiber photometry experiment, viral preparations were injected into the IPN using a Hamilton 10 μL microsyringe (1701RN; Hamilton Company, Reno, NV, USA) and a Harvard Apparatus (PUMP 11 ELITE Nanomite, Harvard, MA, USA). A dual-virus strategy was employed, using rAAV-GAD67-CRE-mCherry-WPRE-hGH polyA and rAAV-EF1 a-DIO-GCaMp6s-WPRE-hGH polyA (virus titers: 5.55E + 12 vg·mL^−1^ and 5.27E + 12 vg·mL^−1^, respectively) (BrainVTA Technology Co., Ltd., Wuhan, China), mixed in a 1:2 ratio and injected at 300 nL per mouse.

In the chemogenetic experiment, 300 nL of rAAV GAD67-hM4D(Gi)-mCherry-WPREs or rAAV GAD67-hM3D(Gq)-mCherry-WPREs (virus titers: 5.27 × 10^12^ vg·mL^−1^ and 6.04 × 10^12^ vg·mL^−1^, respectively) (BrainVTA Technology Co., Ltd., Wuhan, China) was injected into the interpeduncular nucleus (IPN) of C57BL/6 mice. The IPN coordinates were as follows: AP: −3.4 mm; ML: −1.3 mm; DV: −4.85 mm; a 14° angle. After the viral injection, the glass electrode was left in place for 10 min to prevent viral leakage. Optic fibers (Ø1.25 mm/5.5 mm; Thinker Tech Nanjing Bioscience, Nanjing, China) were implanted and secured with dental cement. After surgery, the mice were injected with gentamicin (0.01 mL·10 g^−1^) to prevent infection and were kept in a single cage. The viral injections were performed 3 weeks prior to the start of the experiment to ensure sufficient transgene expression.

### 4.8. Microdialysis Experiment

Prior to implantation, the mouse brain microdialysis probes were soaked in pure water for 30 min and subsequently placed in a mixed standard solution containing glutamate (Glu), D-serine (D-Ser), aspartate (Asp), glycine (Gly), and gamma-aminobutyric acid (GABA). Probes were perfused with saline (1.0 μL·min^−1^) for 30 min to determine the in vitro recovery rates.

On the experimental day, the probes were implanted and perfused with compound sodium chloride (1 μL·min^−1^) for 2 h to achieve equilibration. Baseline dialysate was collected at 30 min intervals over 120 min. Following intraperitoneal ifenprodil administration and the oral gavage of low-/high-dose STZYD, post-administration dialysate was collected continuously for 240 min (12 tubes) under identical conditions. Post-experiment, brains were fixed in 4% tissue fixative, sectioned, and microscopically verified for probe placement, with data included only if the dialysis membrane position deviated ≤ 30% from the target site’s dorsal/ventral boundaries.

### 4.9. Fiber Photometry

The fiber photometry system was provided by Thinker Tech Nanjing Bioscience Inc. A blue excitation light at 470 nm was emitted from an LED source, reflected by a dichroic mirror, and coupled through an objective lens into a multimode optical fiber. The tip of the fiber delivered the excitation light into the IPN region, where it activated GCaMP calcium-sensitive fluorescent proteins expressed in neurons. The light intensity at the fiber tip was maintained at approximately 30 μW to minimize photobleaching. Mechanical and thermal nociceptive stimuli were applied to the left hind paws of mice using a Von Frey filament (0.4 g, Danmic, San Jose, CA, USA) and radiant heat source, respectively, to monitor calcium signal fluctuations in GABAergic neurons within the interpeduncular nucleus (IPN) during pain-related tests. Each stimulus event was time-stamped within the recording window, and the resulting calcium signals were analyzed using MATLAB software (version 2017b; MathWorks, Natick, MA, USA) in conjunction with a fiber photometry analysis package (Thinker Tech Nanjing Bioscience, Nanjing, China). The relative change in the fluorescence intensity (ΔF/F_0_) was calculated by subtracting the baseline fluorescence (F_0_) from the recorded fluorescence (F) and then dividing by F_0_, i.e., (F − F_0_)/F_0_. Here, F_0_ was defined as the mean GCaMP6s signal during the 5 s baseline period immediately preceding either the Von Frey stimulation or the thermal radiant heat stimulus. The fiber photometry data were further processed using custom-written MATLAB scripts. Quantitative parameters, including the area under the curve (AUC) (determined via trapezoidal integration) and peak fluorescence amplitude, were extracted from a 5 s peri-event window, as previously described [64].

### 4.10. Chemogenetic Manipulation

In the chemogenetic experiment, 300 nL of rAAV GAD67-hM4D(Gi)-mCherry-WPREs or rAAV GAD67-hM3D(Gq)-mCherry-WPREs (BrainVTA Technology Co., Ltd., Wuhan, China) was injected into the IPNs of C57BL/6 mice. The IPN coordinates were as follows: AP: −3.4 mm; ML: −1.3 mm; DV: −4.85 mm; a 14° angle. In the chemogenetic inhibition experiment, the hM4D(Gi) vector was injected, and one week later, a SNI model was induced. Behavioral testing was conducted two weeks post-surgery. One hour following the intraperitoneal injection of clozapine-N-oxide (CNO) (5 mg·kg^−1^), the PWT and PWL were measured. In the chemogenetic activation experiment, behavioral testing was performed three weeks after the hM3D(Gq) injection, with CNO (1 mg·kg^−1^) administered one hour prior to the test.

### 4.11. HPLC-FLD Detection Conditions

The ortho-phthalaldehyde (OPA) derivatization reagent was prepared by dissolving 5 mg OPA in 120 μL methanol, followed by the addition of 10 μL β-mercaptoethanol and 1 mL borate buffer (0.2 mol·L^−1^, pH 9.2). The OPA reagent was prepared fresh daily and stored protected from light on ice until use. This solution was mixed thoroughly and stored protected from light. The mobile phase consisted of Liquid A (20 mmol·L^−1^ sodium acetate buffer (pH 7.2)/methanol/tetrahydrofuran at 400:95:5) and Liquid B (buffer/methanol at 120:380). Chromatographic separation was performed at a 0.8 mL·min^−1^ flow rate and 40 °C column temperature using gradient elution: initial column conditioning with 80% methanol/20% water (30 min) followed by 50% methanol/50% water (30 min) and 20% methanol/80% water (30 min). After column pressure stabilization, the analytical gradient was applied: 0–10 min (0–63% B), 10–12 min (63% B), 12–17 min (100% B), 17–18 min (100–0% B), and 18–21 min (0% B). Amino acid analytes underwent automated pre-column derivatization with OPA before injection. Fluorescence detection employed 340 nm excitation and 455 nm emission wavelengths at a photomultiplier tube gain of 12. Blank and sample analyses were conducted following system equilibration.

### 4.12. HPLC-Q-TOF-MS/MS Analysis Conditions

Two hundred microliters of serum or brain homogenate from each mouse was transferred into a 5 mL EP tube, and the proteins were precipitated by adding three volumes of ice-cold methanol. After vortexing for 3 min, samples were centrifuged (8000 rpm, 4 °C, 10 min), the supernatant was dried with nitrogen gas at 40 °C, and then 50% methanol was added to redissolve it. The mixture was vortexed for 3 min and centrifuged at 4 °C and 10,000 r·min^−1^ for 10 min, and 3 μL of the supernatant was injected.

#### 4.12.1. Chromatographic Conditions

Agilent 6520 Q-TOF LC-MS was used for analysis, consisting of an Agilent 1200 liquid chromatograph and an Agilent 6520 Q-TOF time-of-flight mass spectrometer (Agilent Technologies, Santa Clara, CA, USA). For HPLC separation, the sample solution was injected into an ACQUITY UPLC BEH C18 column (2.1 × 100 mm, 1.7 μm, Waters, Milford, MA, USA). The mobile phase consisted of phase A (0.1% formic acid in water) and phase B (ACN), with a linear gradient elution (0–4 min, 95% A; 4–51 min, 95–0% A; 51–56 min, 0% A; 56–58 min, 0–95% A; 58–68 min, 95% A), at a flow rate of 0.4 mL·min^−1^. The column temperature was 40 °C, and the injection volume was 3 μL.

#### 4.12.2. Mass Spectrometric Conditions

The mass spectrometric conditions were as follows: electrospray ionization source (ESI); positive-ion mode; capillary voltage: 3500 V; auto MS/MS mode scanning; scan range: *m*/*z* 50–1750; collision voltages: 10, 20, 40 V; drying gas nitrogen, flow rate: 10 L·min^−1^; nebulizer gas pressure: 40 psi; desolvation gas temperature: 350 °C. All data were processed using MassHunter software (Agilent, v10.0).

### 4.13. Western Blotting

IPN tissue was dissected on a chilled brain matrix, snap-frozen, and lysed in 2% SDS buffer supplemented with protease/phosphatase inhibitor (#78440, Thermo Fisher Scientific Inc., Waltham, MA, USA). Equal amounts of protein were resolved on SDS-PAGE gels and transferred to PVDF membranes. Membranes were blocked with 5% BSA and incubated overnight at 4 °C with the following primary antibodies: NMDAR2B (ab183942, Abcam, Cambridge, UK; 1:1000), CaMKII (ab52476, Abcam, Cambridge, UK; 1:1000), p-CaMKII (#12716, Cell Signaling Technology, Danvers, MA, USA; 1:1000), CREB (#9197, Cell Signaling Technology, Danvers, MA, USA; 1:1000), p-CREB (#9198, Cell Signaling Technology, Danvers, MA, USA; 1:1000), and Vinculin (#26520-1-AP, Proteintech, Rosemont, IL, USA; 1:10,000). After PBST washes, HRP-conjugated secondary antibodies were applied. Protein bands were visualized via chemiluminescence and quantified using ImageJ [65] (version 1.50i, NIH, Bethesda, MD, USA).

### 4.14. mRNA Gene Expression Using RT-qPCR Analysis

Total RNA from IPN punches was extracted with the TRIzol™ Reagent (#15596026, Thermo Fisher Scientific, Waltham, MA, USA) and treated with gDNA Eraser prior to reverse transcription using the PrimeScript™ RT kit (#RR047A, Takara Bio Inc., Shiga, Japan), as detailed by Castellanos-Rizaldos et al. (2020) [66]. The target gene expressions (Table 1) were normalized to GAPDH and quantified using the 2^−ΔΔCt^ method [67].

### 4.15. Immunofluorescence and Immunohistochemistry

For immunofluorescence staining, tissue sections were baked at 65 °C for 1 h and then processed for dewaxing, antigen retrieval, and antibody incubation essentially as described by Wang et al. (2024) [68]: the slides were dewaxed in xylene, rehydrated through a graded ethanol series, and subjected to heat-induced epitope retrieval in 10 mM sodium-citrate buffer (pH 6.0) at 95 °C for 20 min. After blocking with 3% (*w*/*v*) BSA at room temperature for 30 min, the sections were incubated overnight at 4 °C with rabbit anti-c-Fos antibody (ab208942, Abcam, Cambridge, UK; 1:400) and were subsequently incubated for 1 h at room temperature with CoraLite 488-conjugated goat anti-rabbit IgG (H+L) (SA00013-1, Proteintech, Rosemont, IL, USA; 1:400). Nuclei were counterstained with DAPI-containing anti-fade mounting medium (Solarbio S2110) and coverslipped. Fluorescence images were acquired with a KFBIO KF-PRO-020 slide scanner and quantified using ImageJ (version 1.50i, NIH, Bethesda, MD, USA).

For the immunohistochemistry experiment, sections were routinely deparaffinized and subjected to heat-induced antigen retrieval in citrate buffer for 20 min. After blocking for 30 min, the slides were incubated with the primary antibody at room temperature for 2 h, and the appropriate secondary antibody was then applied and left for 30 min at room temperature. Chromogenic detection was performed with DAB, followed by counterstaining with hematoxylin for 5 min. Sections were dehydrated, cleared, mounted, and examined under a microscope. The positive cells were counted using ImageJ software. Primary antibodies used included anti-NMDAR2B (ab183942, Abcam, Cambridge, UK; 1:1000), phospho-CREB (#9198, Cell Signaling Technology, Danvers, MA, USA; 1:1000), and phospho-CaMKII (#12716; Cell Signaling Technology, Danvers, MA, USA; 1:1000).

After the completion of the fiber photometry and chemogenetic experiments, mice were anesthetized with tribromoethanol (0.2 mL·10 g^−1^, i.p.). The brains were removed, post-fixed in 4% PFA for 10 h at 4 °C, and then immersed in a 30% sucrose solution until fully sank. Coronal brain sections (40 μm) were prepared using a cryostat (Epredia™ CryoStar™ NX70, Kalamazoo, MI, USA). Sections were rinsed with PBS, blocked with 0.5% Triton X-100 and 5% BSA for 1 h, and stained with DAPI for nuclear visualization. Viral injection sites were visualized on a confocal microscope (VS120, Olympus, Tokyo, Japan).

### 4.16. Molecular Docking

The molecular docking of four drug-derived molecules in the IPN with the NMDAR-2B receptor was conducted. The protein information was obtained from the UniProt website (https://www.uniprot.org/uniprotkb, accessed on 22 July 2025), and the 3D structure of the target protein was downloaded from the PDB database (https://www.rcsb.org/, accessed on 22 July 2025). Then, hydrogen atoms and charges were added to the protein using Autodock vina 1.5.6, and they were set as the receptors. The files were saved as PDB files for future use. The structures of the drug molecules were obtained from the PubChem database (https://pubchem.ncbi.nlm.nih.gov/, accessed on 22 July 2025), and energy minimization was performed using Discovery Studio 2019. Drug molecule structures were exported as PDB files. Hydrogen atoms and charges were added to the drug molecule structures, and they were set as ligands. The ligand files were exported in PDBQT format. Molecular docking was carried out in Autodock vina 1.5.6, and the results were visually analyzed using PyMol 3.1 and Discovery Studio 2019.

### 4.17. Statistical Analysis

All data represent at least three independent biological replicates, expressed as means ± SEMs. Statistical analyses were conducted using GraphPad Prism (v8.0.1). For the group comparisons, we employed unpaired or paired two-tailed Student’s *t*-tests, one-way ANOVAs, or two-way ANOVAs, as appropriate. Statistical significance was defined as *p* < 0.05 for all analyses. Exact *p*-values for all comparisons are provided in Supplementary Table S2.

## 5. Conclusions

In this study, we demonstrated that Shentong Zhuyu Decoction (STZYD) exerted therapeutic effects on neuropathic pain through a multitarget, multi-pathway mechanism centered on the inhibition of the NMDAR-2B/CaMKII/CREB axis within GABAergic neurons of the IPN. By modulating these neurons, STZYD achieved the precise “circuit editing” of central pain pathways, offering a reversible, adjustable, and non-addictive treatment modality for otherwise refractory conditions such as phantom limb pain and spinal cord injury-induced pain. Qualitative analysis and molecular docking further revealed that licoricesaponin K2, a key component of STZYD, directly bound to NMDAR-2B to block its calcium signaling, thereby preventing central sensitization. These findings lay a solid foundation for the development of CNS-targeted analgesics and advance the paradigm of “precision pain relief”.

## Figures and Tables

**Table 1:** All primers and other sequences used.

Gene	Primer Sequence	Amplicon Size	Gene ID
*GluN1*	Forward Sequence: CCTTTCAGAGCACACTGTGGCT Reverse Sequence: CCAGGAAAACCACATGGCAGAG	164 bp	14810
*GluN2A*	Forward Sequence: CTGCTCCAGTTTGTTGGTGACG Reverse Sequence: CCAGCATGTAGAAAACTCCTGCC	139 bp	14811
*GluN2B*	Forward Sequence: CTGGTGACCAATGGCAAGCATG Reverse Sequence: GGCACAGAGAAGTCAACCACCT	146 bp	14812
*GluA1*	Forward Sequence: CCTACATCGTCACGACTATCCTC Reverse Sequence: AGTTCCACGCAGTAGCCCTCAT	103 bp	14799
*GluA2*	Forward Sequence: TTCCTTGGGTGCCTTTATGCGG Reverse Sequence: CACCATCCTCTCTACAGTCAGG	154 bp	14800
*GluA3*	Forward Sequence: GCAATGACAGCTCATCCTCCGA Reverse Sequence: GCGCTCATTTCCTTCCAGTTGC	110 bp	53623
*GluA4*	Forward Sequence: CGCCTACTCTTGGCAATGACAC Reverse Sequence: GATGCCAAGTCTACACAGTAGCC	151 bp	14802
*GAPDH*	Forward Sequence: ACCCAGAAGACTGTGGATGG Reverse Sequence: TTCAGCTCAGGGATGACCTT	125 bp	14433

**Table 2:** Docking scores of NMDAR-2B with drug molecules in IPN.

Receptor Proteins	Ligands	Binding Energy (kcal·mol^−1^)
NMDAR-2B	L-arginine	−6.2
	Azelaic acid	−5.4
	Formononetin	−7
	Licoricesaponin K2	−8.2
	Senkyunolide F	−7.8
	Lauric acid	−4.3
	L-isoleucine	−5.1

## Data Availability

The original contributions presented in this study are included in the article/Supplementary Material. Further inquiries can be directed to the corresponding author.

## Supplementary Materials

The following supporting information can be downloaded at: https://www.mdpi.com/article/10.3390/ph18101456/s1, Table S1: The MS data of the detected compounds of STZY decoction via HPLC-Q-TOF-MS/MS. Table S2: Summary of statistics. Figure S1: Representative bands of NMDAR-2B, CaMKII, p-CaMKII, CREB and p-CREB.

## Abbreviations

BBB	Blood–brain barrier
CaMKII	Calcium/calmodulin-dependent protein kinase II
CCI	Chronic constriction injury
CNS	Central nervous system
CNO	Clozapine-N-oxide
CREB	cAMP response element-binding protein
DL	Drug-likeness
DREADDs	Designer receptors exclusively activated by designer drugs
EIC	Extracted ion chromatogram
GABA	Gamma-aminobutyric acid
GO	Gene Ontology
HE	Hematoxylin–eosin staining
IPN	Interpeduncular nucleus
KEGG	Kyoto Encyclopedia of Genes and Genomes
MHb	Medial habenula
mPFC	Medial prefrontal cortex
NMDAR	N-methyl-D-aspartate receptor
NMDAR-2B	NMDA receptor subunit 2B
NPP	Neuropathic pain
OB	Oral bioavailability
OPA	Ortho-phthalaldehyde
RT-qPCR	Quantitative real-time PCR
SNI	Spared nerve injury
SNRIs	Serotonin–norepinephrine reuptake inhibitors
STZYD	Shentong Zhuyu Decoction
TCAs	Tricyclic antidepressants
TCM	Traditional Chinese medicine
TIC	Total ion chromatogram
