# Supplementary material for: Shentong Zhuyu Decoction Alleviates Neuropathic Pain in Mice by Inhibiting the NMDAR-2B Receptor-Mediated CaMKII/CREB Signaling Pathway in GABAergic Neurons of the Interpeduncular Nucleus
Source: Pharmaceuticals (Basel). 2025 Sep 28;18(10):1456. doi: 10.3390/ph18101456 (PMC12567535; doi:10.3390/ph18101456)
Supplement: Supplementary file 1 [file pharmaceuticals-18-01456-s001.zip › Table S1 The MS data of detected compounds of STZY decoction by HPLC-Q-TOF-MSMS..docx]

| **No.** | **Identification** | **RT (min)** | **Molecular Formula** | **Calculated** | **Observed** | **Ion mode** | **Error(ppm)** | **MS/MS fragments** | **Distribution** | **Source** |
| --- | --- | --- | --- | --- | --- | --- | --- | --- | --- | --- |
| 1 | L-arginine | 0.791 | C_6_H_14_N_4_O_2_ | 175.1190 | 175.1191 | [M+H]^+^ | 0.69 | 175.1191,158.0918,130.0973,116.0704,70.0656 | a,c | TR、CQ、NX |
| 2 | Cytosine | 0.907 | C_4_H_5_N_3_O | 112.0506 | 112.0499 | [M+H]^+^ | -5.89 | 112.0499,94.9884,83.0483,70.0650,55.0542 | a | TR |
| 3 | Betaine | 0.907 | C_5_H_11_NO_2_ | 118.0863 | 118.0855 | [M+H]^+^ | -6.61 | 118.0850,72.0808,59.0734,55.0547 | a | QH |
| 4 | L-Valine | 0.907 | C_5_H_11_NO_2_ | 118.0863 | 118.0863 | [M+H]^+^ | 0.17 | 118.0863,72.0808,59.0734,58.0657,55.0547 | a | TR |
| 5 | L-Pyroglutamicacid | 1.023 | C_5_H_7_NO_3_ | 128.0353 | 128.0352 | [M-H]^-^ | -0.70 | 128.0349,111.0231,99.9282,85.0304,71.0123,57.0334 | a,b | DL |
| 6 | L-Malic acid | 1.023 | C_4_H_6_O_5_ | 133.0142 | 133.0141 | [M-H]^-^ | -0.90 | 133.0144,115.0038,92.0246,71.0147,59.0167 | a | CX、HH |
| 7 | Hypoxanthine | 1.023 | C_5_H_4_N_4_O | 137.0458 | 137.0463 | [M+H]^+^ | 3.58 | 137.0463,118.9222,81.9377,68.9980,56.9658 | a | WLZ |
| 8 | Citric Acid | 1.023 | C_6_H_8_O_7_ | 191.0197 | 191.0198 | [M-H]^-^ | 0.52 | 191.0198,173.0065,111.0082,87.0082,57.0355 | a | TR、HH |
| 9 | Adenosine | 1.023 | C_10_H_13_N_5_O_4_ | 268.1041 | 268.1043 | [M+H]^+^ | 0.93 | 268.1043,253.1722,193.1014,136.0611,73.0298 | a | TR |
| 10 | L-Tyrosine | 1.255 | C_9_H_11_NO_3_ | 180.0666 | 180.0665 | [M-H]^-^ | -0.50 | 180.0665,135.0457,126.0111,108.0462 | a | TR、WLZ |
| 11 | L-Isoleucine | 1.4865 | C_6_H_13_NO_2_ | 132.1019 | 132.1018 | [M+H]^+^ | -0.98 | 132.1018,106.0655,86.0970,74.0232 | a | DL、CX、QH |
| 12 | D-Pantothenic acid | 2.647 | C_9_H_17_NO_5_ | 220.1180 | 220.1183 | [M+H]^+^ | 1.50 | 220.1181,202.1080,184.0966,90.0554 | a | NX |
| 13 | Protocatechuic acid | 2.7005 | C_7_H_6_O_4_ | 155.0339 | 155.0334 | [M+H]^+^ | -3.29 | 155.0342,137.0232,116.9762,93.0331,65.0392 | a,b | WLZ、CX |
| 14 | 1,2-Benzenediol | 2.877 | C_6_H_6_O_2_ | 109.0295 | 109.0297 | [M-H]^-^ | 2.02 | 109.0293,91.0191,81.0344,68.9962,53.0406 | a,b | WLZ |
| 15 | Neochlorogenic acid | 3.687 | C_16_H_18_O_9_ | 355.1024 | 355.1029 | [M+H]^+^ | 1.46 | 355.1029,163.0390,145.0285 | a | CX |
| 16 | 3-Indoleacrylic acid | 4.035 | C_11_H_9_NO_2_ | 188.0706 | 188.0704 | [M+H]^+^ | -1.22 | 188.0708,170.0592,146.0601,118.0648 | a | NX、QH  Continued |
| 17 | Indole-3-carbaldehyde | 4.151 | C_9_H_7_NO | 146.0601 | 146.0601 | [M+H]^+^ | 0.27 | 146.0596,132.0648,118.0648,91.0546 | a | DG |
| **No.** | **Identification** | **RT (min)** | **Molecular Formula** | **Calculated** | **Observed** | **Ion mode** | **Error(ppm)** | **MS/MS fragments** | **Distribution** | **Source** |
| 18 | DL-Tryptophan | 4.2085 | C_11_H_12_N_2_O_2_ | 205.0972 | 205.0983 | [M+H]^+^ | 5.46 | 205.0983,149.0235,89.0596,57.0697 | a | NX、DG |
| 19 | Indole-3-acetic acid | 4.266 | C_11_H_9_NO_2_ | 188.0706 | 188.0707 | [M+H]^+^ | 0.37 | 188.0707,146.0601,118.0648,87.0400 | a | DG |
| 20 | 3-Hydroxybenzoicacid | 4.499 | C_7_H_6_O_3_ | 137.0244 | 137.0241 | [M-H]^-^ | -2.12 | 137.0234,93.0341,65.0399 | a,b | WLZ |
| 21 | Desaminotyrosine | 4.962 | C_9_H_10_O_3_ | 165.0557 | 165.056 | [M-H]^-^ | 1.88 | 165.0560,141.8644,121.0653,93.0340,59.0144 | a,b | HH |
| 22 | Ferulic acid | 6.467 | C_10_H_10_O_4_ | 195.0652 | 195.0653 | [M+H]^+^ | 0.46 | 196.0661,177.0542,145.0280 | a,b | QH、QJ |
| 23 | Chlorogenic acid | 7.047 | C_16_H_18_O_9_ | 355.1024 | 355.1034 | [M+H]^+^ | 2.87 | 355.1028,163.0389 | a | QH、CX |
| 24 | Caffeic acid 1 | 7.394 | C_9_H_8_O_4_ | 181.0496 | 181.0494 | [M+H]^+^ | -0.88 | 181.0498,163.0386,145.0281,135.0435,117.0341 | a | CX |
| 25 | Caffeic acid 2 | 7.394 | C_9_H_8_O_4_ | 181.0496 | 181.0493 | [M+H]^+^ | -1.44 | 181.0509,163.0389,135.0435,109.0628 | a | CX |
| 26 | Salicylic acid | 7.511 | C_7_H_6_O_3_ | 137.0244 | 137.0242 | [M-H]^-^ | -1.39 | 137.0237,119.0124,108.0211,92.0260,81.0335 | a,b | XF |
| 27 | Hydroxysafflor Yellow A | 7.7425 | C_27_H_32_O_16_ | 613.1763 | 613.177 | [M+H]^+^ | 1.08 | 613.1744,451.1228,433.1118,415.1022,331.0816 | a,b | HH |
| 28 | Cryptochlorogenic acid | 7.743 | C_16_H_18_O_9_ | 353.0878 | 353.0881 | [M-H]^-^ | 0.91 | 353.0863,191.0548 | a | XF、CX |
| 29 | Vanillic acid | 7.861 | C_8_H_8_O_4_ | 167.0350 | 167.0341 | [M-H]^-^ | -5.15 | 167.0344,123.0444,79.0551 | a | DG、CX |
| 30 | D-Amygdalin | 8.091 | C_20_H_27_NO_11_ | 456.1511 | 456.1518 | [M-H]^-^ | 1.51 | 456.1519,323.0988,263.0765,221.0645,179.0560 | a,b | TR |
| 31 | Fraxin | 8.438 | C_16_H_18_O_10_ | 369.0827 | 369.0836 | [M-H]^-^ | 2.44 | 369.0824,354.0688,207.0294,192.0053 | a | QH |
| 32 | Sweroside | 8.787 | C_16_H_22_O_9_ | 359.1337 | 359.1348 | [M+H]^+^ | 3.12 | 359.1335,197.0803,127.0385 | a,b | QJ |
| 33 | Scutellarin | 8.787 | C_22_H_32_O_14_ | 521.1865 | 521.1871 | [M+H]^+^ | 1.13 | 521.1871,359.1335,197.0803,127.0385 | a | QJ |
| 34 | Neoisoliquiritin | 8.9 | C_21_H_22_O_9_ | 419.1337 | 419.1338 | [M+H]^+^ | 0.29 | 419.1349,257.0809,137.0237 | a | GC |
| 35 | Saponarin | 9.135 | C_27_H_30_O_15_ | 595.1658 | 595.1653 | [M+H]^+^ | -0.79 | 595.1684,391.1586,244.1002,193.0441,127.0335 | a | QJ |
| 36 | （R）-Prunasin | 9.249 | C_14_H_17_NO_6_ | 294.0983 | 294.0986 | [M-H]^-^ | 1.05 | 294.0986,188.0565,161.0489,101.0245,71.0144 | a,b | TR |
| **No.** | **Identification** | **RT (min)** | **Molecular Formula** | **Calculated** | **Observed** | **Ion mode** | **Error(ppm)** | **MS/MS fragments** | **Distribution** | **Source** |
| 37 | α-Lactose monohydrate | 9.249 | C_12_H_22_O_11_ | 341.1089 | 341.1098 | [M-H]^-^ | 2.61 | 341.1098,295.1025,188.0558,161.0451,101.0245 | a,b | TR |
| 38 | p-Coumaric acid | 9.829 | C_9_H_8_O_3_ | 163.0400 | 163.0405 | [M-H]^-^ | 2.82 | 163.0403,119.0498,103.0043,87.0092,59.0145 | a,b | NX、CX、QH |
| 39 | Vicenin-2 | 9.943 | C_27_H_30_O_15_ | 595.1658 | 595.1651 | [M+H]^+^ | -1.13 | 595.1653,495.6082,433.1124,313.0547,209.0474 | a | GC |
| 40 | Ginkgolide | 10.524 | C_15_H_18_O_8_ | 325.0929 | 325.0927 | [M-H]^-^ | -0.52 | 325.0927,283.0981,235.0981,147.0453,119.0512,71.0148 | a,b | HH |
| 41 | Schaftoside | 10.754 | C_26_H_28_O_14_ | 565.1552 | 565.1555 | [M+H]^+^ | 0.52 | 565.1551,547.1446,481.1112,379.0803,325.0710 | a | GC |
| 42 | Luteolin-6-C-glucoside | 10.837 | C_21_H_20_O_11_ | 449.1079 | 449.1086 | [M+H]^+^ | 1.65 | 449.1086,431.0952,383.0817,329.0634 | a | QJ |
| 43 | Scopoletin | 10.87 | C_10_H_8_O_4_ | 193.0496 | 193.0499 | [M+H]^+^ | 1.76 | 193.0496,178.0260,165.0547,133.0284 | a,b | QH |
| 44 | Polypodine B | 10.8740 | C_27_H_44_O_8_ | 497.3109 | 497.3125 | [M+H]^+^ | 3.18 | 497.3105,461.2883,371.2223,303.1969,249.1490 | a | NX |
| 45 | Methoxycoumarin | 11.101 | C_10_H_8_O_3_ | 177.0546 | 177.0545 | [M+H]^+^ | -0.79 | 177.0545,145.0285,117.0331,89.0367,57.0723 | a,b | DG |
| 46 | Isoferulic acid | 11.103 | C_10_H_10_O_4_ | 193.0506 | 193.0510 | [M-H]^-^ | 2.02 | 193.0526,178.0273,134.0378,102.0110,85.0309 | a,b | XF、CX、DG |
| 47 | Achyranthesterone A | 11.106 | C_27_H_44_O_8_ | 497.3109 | 497.3102 | [M+H]^+^ | -1.45 | 497.3115,479.2996,443.2797,387.2164,303.1952 | a | NX |
| 48 | Marmesinin | 11.565 | C_20_H_24_O_9_ | 409.1493 | 409.1502 | [M+H]^+^ | 2.13 | 409.1497,247.0966,229.0857,187.0385,85.0285 | a | QH |
| 49 | Violanthin | 11.799 | C_27_H_30_O_14_ | 579.1709 | 579.1698 | [M+H]^+^ | -1.82 | 579.1682,419.1330,257.0804,137.0230 | a | GC |
| 50 | Glycyrrhizin-Glucosyluronic acid | 11.799 | C_27_H_30_O_14_ | 579.1709 | 579.1698 | [M+H]^+^ | -1.83 | 579.1682,419.1330,257.0804,137.0230 | a | GC |
| 51 | Liquiritin | 11.8555 | C_21_H_22_O_9_ | 419.1337 | 419.1344 | [M+H]^+^ | 1.72 | 419.1346,257.0806,137.0230 | a | GC |
| 52 | Glycyrrhizin-4'-O-rutinoside | 11.917 | C_26_H_30_O_13_ | 549.1613 | 549.1628 | [M-H]^-^ | 2.66 | 549.1628,417.1230,255.0673,135.0090 | a,b | GC |
| **No.** | **Identification** | **RT (min)** | **Molecular Formula** | **Calculated** | **Observed** | **Ion mode** | **Error(ppm)** | **MS/MS fragments** | **Distribution** | **Source** |
| 53 | Vitexin | 12.034 | C_21_H_20_O_10_ | 433.1130 | 433.1131 | [M+H]^+^ | 0.35 | 433.1129,415.1012,313.0699,283.0606,257.0759 | a,b | QJ |
| 54 | Benzoic acid | 12.1475 | C_7_H_6_O_2_ | 123.0441 | 123.0443 | [M+H]^+^ | 1.79 | 123.0442,105.0342,95.9536,81.9378,79.0547 | a,b | TR |
| 55 | Liquiritigenin | 12.208 | C_15_H_12_O_4_ | 257.0809 | 257.0812 | [M+H]^+^ | 1.32 | 257.0812,239.0701,147.0439,137.0233 | a | GC |
| 56 | Nodakenin | 12.726 | C_20_H_24_O_9_ | 409.1493 | 409.1503 | [M+H]^+^ | 2.37 | 409.1494,247.0962,229.0857,187.0382 | a,b | QH |
| 57 | Senkyunolide G | 12.842 | C_12_H_16_O_3_ | 209.1172 | 209.1171 | [M+H]^+^ | -0.67 | 209.1171,191.1066,163.1115,153.0545,121.1011 | a,b | CX |
| 58 | Nicotiflorin | 12.8425 | C_27_H_30_O_15_ | 595.1658 | 595.1647 | [M+H]^+^ | -1.80 | 595.1647,533.0942,433.1093,287.0593,147.0625 | a | HH |
| 59 | Senkyunolide J/N | 12.952 | C_12_H_18_O_4_ | 227.1278 | 227.1276 | [M+H]^+^ | -0.92 | 227.1271,209.1171,191.1062,153.0548,135.1169 | a,b | CX |
| 60 | Stachysterone | 12.958 | C_27_H_42_O_6_ | 463.3054 | 463.3053 | [M+H]^+^ | -0.30 | 463.3053,445.2957,301.1796,197.1529,81.0684 | a | NX |
| 61 | Inokosterone A | 12.958 | C_27_H_44_O_7_ | 481.3160 | 481.3164 | [M+H]^+^ | 0.83 | 481.3169,463.3048,445.2960,428.2856,197.1531 | a | NX |
| 62 | 1,5-Dicaffeoylquinic acid | 13.074 | C_25_H_24_O_12_ | 517.1341 | 517.1344 | [M+H]^+^ | 0.62 | 517.1344,499.1246,337.0930,207.1014,163.0389 | a | CX、QH |
| 63 | Commiphoric acid | 13.075 | C_15_H_20_O_4_ | 263.1289 | 263.1292 | [M-H]^-^ | 1.29 | 263.1292,219.1371,149.0953,125.0231,59.0144 | a,b | MY |
| 64 | Isorhamnetin 3-O-neohesperidoside | 13.185 | C_28_H_32_O_16_ | 625.1763 | 625.176 | [M+H]^+^ | -0.54 | 625.1760,369.1216,209.0926 | a | HH |
| 65 | Chrysoeriol | 13.421 | C_16_H_12_O_6_ | 301.0707 | 301.0706 | [M+H]^+^ | -0.29 | 301.0715,286.0459,261.0770 | a | GC |
| 66 | 6'-Acetylglycyrrhizin | 13.653 | C_24_H_24_O_12_ | 505.1341 | 505.1338 | [M+H]^+^ | -0.55 | 505.1325,385.0754,257.0805,137.0234,85.0289 | a | GC |
| 67 | Azelaic acid | 13.655 | C_9_H_16_O_4_ | 187.0976 | 187.0983 | [M-H]^-^ | 3.96 | 187.0983,146.9354,125.0960,102.9479 | a,b | DL |
| 68 | Senkyunolide D | 13.878 | C_12_H_14_O_4_ | 223.0965 | 223.0975 | [M+H]^+^ | 4.44 | 223.0948,209.1169,191.1069,153.0551,135.1149 | a | CX |
| **No.** | **Identification** | **RT (min)** | **Molecular Formula** | **Calculated** | **Observed** | **Ion mode** | **Error(ppm)** | **MS/MS fragments** | **Distribution** | **Source** |
| 69 | Podecdysone B | 14 | C_28_H_46_O_7_ | 495.3317 | 495.3311 | [M+H]^+^ | -1.11 | 495.3310,477.3200,459.3083,441.3001,211.1686 | a | NX |
| 70 | 1,2,8-Trihydroxy-6-methoxyxanthone | 14.119 | C_14_H_10_O_6_ | 275.0550 | 275.0561 | [M+H]^+^ | 3.85 | 275.0562,257.0443,121.0286,85.0289 | a | QJ |
| 71 | Senkyunolide F | 14.464 | C_12_H_14_O_3_ | 207.1016 | 207.1017 | [M+H]^+^ | 0.53 | 207.1014,189.0909,161.0948,119.0842,91.0535 | a,b | CX |
| 72 | Senkyunolide I | 14.464 | C_12_H_16_O_4_ | 225.1122 | 225.1117 | [M+H]^+^ | -2.04 | 225.1133,207.1031,189.0899,165.0913,137.0494,119.0856 | a,b | CX |
| 73 | 7,4'-Dihydroxyflavone | 14.694 | C_15_H_10_O_4_ | 255.0652 | 255.0660 | [M+H]^+^ | 3.10 | 255.0656,147.0429,137.0234,107.0476 |  | GC |
| 74 | Liquiritin apioside | 14.696 | C_26_H_30_O_13_ | 551.1759 | 551.1763 | [M+H]^+^ | 0.65 | 551.1752,419.1332,257.0804,133.0491 | a | GC |
| 75 | Liquiritin | 14.986 | C_21_H_22_O_9_ | 419.1337 | 419.1346 | [M+H]^+^ | 2.20 | 419.1330,257.0806,137.0230 | a | GC |
| 76 | Ononin | 15.275 | C_22_H_22_O_9_ | 431.1337 | 431.1338 | [M+H]^+^ | 0.27 | 431.1348,269.0807 | a,b | GC |
| 77 | Licochalcone B | 15.39 | C_16_H_14_O_5_ | 287.0914 | 287.0923 | [M+H]^+^ | 3.07 | 287.0906,245.0815,193.0500,151.0389,121.0279 | a | GC |
| 78 | N-trans-Feruloyltyramine | 15.39 | C_18_H_19_NO_4_ | 314.1387 | 314.1380 | [M+H]^+^ | -2.26 | 314.1382,177.0542,145.0282,121.0651 | a | NX |
| 79 | Isoliquiritigenin | 15.6785 | C_15_H_12_O_4_ | 257.0809 | 257.0809 | [M+H]^+^ | 0.16 | 257.0809,239.0702,147.0440,137.0232 | a | GC |
| 80 | N-trans-Feruloyl-3-methoxytyramine | 15.854 | C_19_H_21_NO_5_ | 344.1493 | 344.1501 | [M+H]^+^ | 2.41 | 344.1493,177.0542,145.0278 | a | NX |
| 81 | Luteolin | 16.086 | C_15_H_10_O_6_ | 287.0550 | 287.0552 | [M+H]^+^ | 0.56 | 287.0537,235.1680,193.1200,155.1072,119.0895 | a | XF、QJ |
| 82 | Myrrhterpenoid K | 16.201 | C_15_H_18_O_3_ | 247.1329 | 247.1330 | [M+H]^+^ | 0.45 | 247.1330,229.1213,201.1268,139.0387,107.0853 | a | MY |
| 83 | Myrrhanoperoxide | 16.551 | C_15_H_20_O_4_ | 263.1289 | 263.1282 | [M-H]^-^ | -2.51 | 263.1282,219.1395,163.1129,95.0505 | a,b | MY |
| 84 | 6'-Feruloylnodakenin | 17.128 | C_30_H_32_O_12_ | 585.1967 | 585.1979 | [M+H]^+^ | 2.08 | 585.1942,421.3482,339.1079,247.0952,177.0540 | a | QH |
| 85 | Myrrhterpenoid C | 17.13 | C_16_H_22_O_4_ | 277.1445 | 277.1455 | [M-H]^-^ | 3.57 | 277.1455,261.1123,237.1498,217.1245,201.1290 | a | MY |
| **No.** | **Identification** | **RT (min)** | **Molecular Formula** | **Calculated** | **Observed** | **Ion mode** | **Error(ppm)** | **MS/MS fragments** | **Distribution** | **Source** |
| 86 | Uralsaponin P | 17.36 | C_42_H_64_O_16_ | 825.4267 | 825.4268 | [M+H]^+^ | 0.08 | 825.4250,649.3933,455.3512, | a | GC |
| 87 | 3-Butylidenephthalide | 17.476 | C_12_H_12_O_2_ | 189.0910 | 189.0913 | [M+H]^+^ | 1.43 | 189.0908,171.0800,161.0957,128.0617,105.0334 | a | CX、DG |
| 88 | Alismol | 17.592 | C_15_H_24_O | 221.1900 | 221.1900 | [M+H]^+^ | -0.09 | 221.1902,203.1786,135.1164,81.0702,71.0490 | a | MY |
| 89 | Myrrhanolide B | 17.708 | C_15_H_18_O_4_ | 263.1278 | 263.1286 | [M+H]^+^ | 3.00 | 263.1279,245.1173,217.1222,189.1271,147.0799 | a,b | MY |
| 90 | Commiphoranoid A | 17.709 | C_14_H_18_O_2_ | 217.1234 | 217.1229 | [M-H]^-^ | -2.21 | 217.1229,202.0982,190.0156,175.1127 | a,b | MY |
| 91 | Echinatin | 18.055 | C_16_H_14_O_4_ | 271.0965 | 271.0967 | [M+H]^+^ | 0.70 | 271.0967,229.0858,177.0542,121.0282 | a | GC |
| 92 | Uralsaponin F | 18.171 | C_44_H_64_O_19_ | 897.4115 | 897.4120 | [M+H]^+^ | 0.58 | 897.4110,703.3673,527.3367,317.0493,159.0287 | a | GC |
| 93 | Licoricesaponin H_2_ | 18.1730 | C_42_H_62_O_16_ | 821.3965 | 821.3955 | [M-H]^-^ | -1.21 | 821.3955,351.0571 | a | GC |
| 94 | 2-Hydroxyglycyrrhetinic acid | 18.4035 | C_42_H_62_O_18_ | 855.4009 | 855.4035 | [M+H]^+^ | 3.02 | 855.4035,679.3697,485.3268,159.0288 | a | GC |
| 95 | Commiphorene B | 18.75 | C_15_H_16_O_3_ | 245.1172 | 245.1175 | [M+H]^+^ | 1.06 | 245.1169,229.1219,201.1263,121.1006 | a | MY |
| 96 | Licoricesaponin A_3_ | 18.924 | C_48_H_72_O_21_ | 985.4639 | 985.4642 | [M+H]^+^ | 0.29 | 985.4667,809.4356,615.3911,453.3374 | a | GC |
| 97 | Commiphorane E_3_ | 18.925 | C_15_H_22_O_3_ | 251.1642 | 251.1649 | [M+H]^+^ | 2.83 | 251.1642,233.1533,215.1426,187.1482,151.0745 | a,b | MY |
| 98 | Myrrhterpenoid H | 18.984 | C_16_H_20_O_4_ | 275.1289 | 275.1295 | [M-H]^-^ | 2.33 | 275.1295,243.1013,228.0773,213.0536 | a | MY |
| 99 | 22-Acetoxyl-glycyrrhizin | 19.445 | C_44_H_64_O_18_ | 881.4166 | 881.4156 | [M+H]^+^ | -1.10 | 881.4177,705.3854,529.3532,511.3428, | a | GC |
| 100 | Glycyrrhizin | 19.677 | C_42_H_60_O_17_ | 837.3904 | 837.3908 | [M+H]^+^ | 0.54 | 837.3887,663.3730,469.3310 | a | GC |
| 101 | Licoricesaponin G | 19.85 | C_42_H_62_O_17_ | 839.4060 | 839.4060 | [M+H]^+^ | 0.00 | 839.4039,663.3722,469.3306,353.0729 | a | GC |
| 102 | Atractylenolide III | 19.996 | C_15_H_20_O_3_ | 249.1485 | 249.1489 | [M+H]^+^ | 1.44 | 249.1485,231.1378,177.0910,121.1010 | a,b | MY |
| 103 | 3-Butylidenephthalide | 20.14 | C_12_H_12_O_2_ | 189.0910 | 189.0912 | [M+H]^+^ | 0.90 | 189.0906,171.0800,147.0436,133.0278,117.0695 | a,b | CX |
| **No.** | **Identification** | **RT (min)** | **Molecular Formula** | **Calculated** | **Observed** | **Ion mode** | **Error(ppm)** | **MS/MS fragments** | **Distribution** | **Source** |
| 104 | Commiterpene D | 20.256 | C_18_H_22_O_5_ | 319.1540 | 319.1540 | [M+H]^+^ | -0.06 | 319.1532,303.2313,263.1262,227.1054,155.1058 | a | MY |
| 105 | Ligustilide | 20.372 | C_12_H_14_O_2_ | 191.1067 | 191.1067 | [M+H]^+^ | 0.10 | 191.1065,163.1116,149.0593,135.0440,105.0697 | a,b | CX、DG |
| 106 | 3-Butylphthalide | 20.372 | C_12_H_14_O_2_ | 191.1067 | 191.1067 | [M+H]^+^ | 0.10 | 191.1067,173.0959,149.0594,135.0434,117.0692 | a | DG |
| 107 | Formononetin | 20.372 | C_16_H_12_O_4_ | 269.0809 | 269.0811 | [M+H]^+^ | 0.90 | 269.0811,254.0569,237.0546,136.0151,118.0409 | a,b,c | GC |
| 108 | (Z)-5,8,11 Trihydroxyoctadec-9-enoic acid | 20.49 | C_18_ H_34_ O_5_ | 329.2333 | 329.2335 | [M-H]^-^ | 0.55 | 329.2335,229.1443,211.1333,171.1008 | a | DG |
| 109 | Licoricesaponin E_2_ | 21.069 | C_42_H_60_O_16_ | 819.3808 | 819.3818 | [M-H]^-^ | 1.17 | 819.3799,351.0573,193.0337,113.0237 | a | GC |
| 110 | β-Asarone | 21.299 | C_15_H_22_O_2_ | 235.1693 | 235.1697 | [M+H]^+^ | 1.79 | 235.1692,217.1584,199.1479,175.1477,159.1164 | a | XF |
| 111 | 22-bAcetoxylglyrrhaldehyde | 21.53 | C_44_H_64_O_17_ | 865.4217 | 865.4200 | [M+H]^+^ | -1.91 | 865.4175,689.3903,495.3462,453.3321,84.9963 | a | GC |
| 112 | Commiphoins A | 21.533 | C_15_H_18_O_4_ | 261.1132 | 261.1142 | [M-H]^-^ | 3.79 | 261.1124,217.1230,175.1119,149.0966,83.0504 | a,b | MY |
| 113 | Commiphoranoid C | 21.995 | C_20_H_28_O_4_ | 333.2061 | 333.2055 | [M+H]^+^ | -1.68 | 333.2055,274.2230,208.0240,190.0108,125.9772,89.4422 | a | MY |
| 114 | Myrrhone | 22.041 | C_15_H_16_O_2_ | 229.1223 | 229.1216 | [M+H]^+^ | -3.19 | 229.1216,211,1110,187.1109,159.0798,69.0338 | a,b | MY |
| 115 | Myrrhterpenoid J | 22.225 | C_16_H_20_O_3_ | 261.1485 | 261.1489 | [M+H]^+^ | 1.38 | 261.1481,229.1219,187.1111,159.0799,85.0647 | a | MY |
| 116 | Glycyrrhetinic acid Monoglucuronide | 22.225 | C_36_H_54_O_10_ | 647.3790 | 647.3801 | [M+H]^+^ | 1.70 | 647.3795,471.3466,453.3370 | a | GC |
| 117 | Licoricesaponin K_2_ | 22.2265 | C_42_H_62_O_16_ | 823.4111 | 823.4136 | [M+H]^+^ | 3.05 | 823.4121,647.3804,471.3464,453.3365 | a | GC |
| 118 | Commiphoin C | 22.341 | C_15_H_16_O_3_ | 245.1172 | 245.1178 | [M+H]^+^ | 2.28 | 245.1179,227.1071,203.1062,175.0752 | a | MY |
| **No.** | **Identification** | **RT (min)** | **Molecular Formula** | **Calculated** | **Observed** | **Ion mode** | **Error(ppm)** | **MS/MS fragments** | **Distribution** | **Source** |
| 119 | Myrrhterpenoid F | 22.341 | C_17_H_20_O_4_ | 289.1435 | 289.1448 | [M+H]^+^ | 4.63 | 289.1448,229.1225,201.1274,159.0812,69.0345 | a | MY |
| 120 | Senkyunolide M | 22.689 | C_16_H_22_O_4_ | 279.1591 | 279.1597 | [M+H]^+^ | 2.11 | 279.1590,233.1540,191.1068,149.0236,105.0331 | a | CX |
| 121 | 1,2-Benzenedicarboxylic acid | 22.689 | C_16_ H_22_ O_4_ | 279.1591 | 279.1589 | [M+H]^+^ | -0.75 | 279.1589,233.1534,191.1059,149.0222,71.0498 | a | DG |
| 122 | Licorice-saponin B_2_ | 23.152 | C_42_H_64_O_15_ | 809.4318 | 809.4330 | [M+H]^+^ | 1.46 | 809.4301,633.3989,439.3565,353.0736,159.0272 | a | GC |
| 123 | Licoricesaponin J2 | 23.387 | C_42_H_62_O_16_ | 821.3965 | 821.3960 | [M-H]^-^ | -0.60 | 821.3960,759.3922,351.0573,289.0560 | a | GC |
| 124 | Licoisoflavone B | 23.618 | C_20_H_16_O_6_ | 353.1020 | 353.1024 | [M+H]^+^ | 1.17 | 353.1024,311.0556,179.0334,151.0387 | a | GC |
| 125 | Alismin | 23.966 | C_15_H_22_O_2_ | 235.1693 | 235.1695 | [M+H]^+^ | 0.94 | 235.1699,217.1592,161.0961,85.0651 | a | MY |
| 126 | Senkyunolide A | 24.082 | C_12_H_16_O_2_ | 193.1223 | 193.1228 | [M+H]^+^ | 2.43 | 193.1225,175.1117,147.1167,137.0608,105.0702 | a,b | CX |
| 127 | Uralsaponin C | 24.082 | C_42_H_64_O_16_ | 825.4267 | 825.4254 | [M+H]^+^ | -1.62 | 825.4286,613.3754,353.0715,159.0289 | a | GC |
| 128 | Glycycoumarin | 24.891 | C_21_H_20_O_6_ | 369.1333 | 369.1342 | [M+H]^+^ | 2.47 | 369.1337,313.0704,285.0757 | a | GC |
| 129 | Isolicoflavonol | 25.702 | C_20_H_18_O_6_ | 355.1176 | 355.1185 | [M+H]^+^ | 2.42 | 355.1178,299.0549,287.0549,69.0701 | a | GC |
| 130 | Commiphorane E_2_ | 26.051 | C_16_H_18_O_3_ | 259.1329 | 259.1335 | [M+H]^+^ | 2.35 | 259.1328,229.1218,205.0852,177.0894,95.0496 | a | MY |
| 131 | Commiphoin B | 27.325 | C_15_H_16_O | 213.1274 | 213.1270 | [M+H]^+^ | -1.97 | 213.1270,185.1318,157.1006,107.0854 | a | MY |
| 132 | 9,10-Dihydroxy-12(Z)-octadecenoic Acid | 27.445 | C_18_ H_34_ O_4_ | 313.2384 | 313.2381 | [M-H]^-^ | -0.99 | 313.2381,295.2315,201.1128 | a | DG |
| 133 | Gancaonin M | 28.484 | C_20_H_16_O_6_ | 353.1020 | 353.1028 | [M+H]^+^ | 2.29 | 353.1031,311.0553 | a | GC |
| 134 | Glyasperin A | 32.422 | C_25_H_26_O_6_ | 423.1802 | 423.1814 | [M+H]^+^ | 2.74 | 423.1814,367.1198,311.0567 | a | GC |
| 135 | Ursolic Acid | 32.426 | C_30_H_48_O_3_ | 457.3677 | 457.3685 | [M+H]^+^ | 1.86 | 457.3685,439.3586,237.1826,95.0871 | a | QJ |
| 136 | Lauric acid | 34.16 | C_12_H_24_O_2_ | 201.1849 | 201.1858 | [M+H]^+^ | 4.32 | 201.1858,149.0230,95.0865,71.0862,57.0711 | a | DL |
| 137 | Tokinolide B | 34.276 | C_24_ H_28_ O_4_ | 381.2061 | 381.2077 | [M+H]^+^ | 4.30 | 381.2077,363.1971,335.2021,191.1071 | a | DG |
| **No.** | **Identification** | **RT (min)** | **Molecular Formula** | **Calculated** | **Observed** | **Ion mode** | **Error(ppm)** | **MS/MS fragments** | **Distribution** | **Source** |
| 138 | 13-hydroxy-9,11-octadecadienoic acid | 34.971 | C_18_H_32_O_3_ | 297.2425 | 297.2437 | [M+H]^+^ | 4.21 | 297.2425,279.2324,261.2215,243.2112,171.1018 | a | CX、DG、NX |
| 139 | Oleanic acid | 37.758 | C_30_H_48_O_3_ | 457.3677 | 457.3680 | [M+H]^+^ | 0.77 | 457.3674,439.3558,427.3551,237.1832,95.0877 | a | QJ |
| 140 | Oleamide | 40.071 | C_18_H_35_NO | 282.2792 | 282.2801 | [M+H]^+^ | 3.29 | 282.2801,265.2519,170.1544,111.1159,83.0859 | a | DL |
| 141 | Linoleic acid | 40.8845 | C_18_H_32_O_2_ | 281.2475 | 281.2476 | [M+H]^+^ | 0.25 | 281.2465,263.2378,245.2262,97.1014 | a,b | NX、DG |
| 142 | Stearic acid amide | 43.546 | C_18_ H_37_ NO | 284.2948 | 284.2949 | [M+H]^+^ | 0.28 | 284.2949,186.1861,116.1075,88.0760,57.0708 | a | DG、DL |
| 143 | Oleic acid | 43.778 | C_18_H_34_O_2_ | 283.2632 | 283.2640 | [M+H]^+^ | 2.89 | 283.2640,265.2513,247.2419,71.0865,57.0706 | a | TR |
| 144 | Myrrhterpenoid A | 46.79 | C_18_H_20_O_4_ | 301.1435 | 301.1422 | [M+H]^+^ | -4.18 | 301.1428,171.8234,89.0604 | a | MY |
| 145 | Lupenone | 49.109 | C_30_H_48_O | 425.3778 | 425.3780 | [M+H]^+^ | 0.42 | 425.3776,329.2854,231.2098,177.1626,109.1005 | a | MY |

**Distribution:** Compound formula: a; Plasma: b; IPN: c
