# Supplementary material for: Shentong Zhuyu Decoction Alleviates Neuropathic Pain in Mice by Inhibiting the NMDAR-2B Receptor-Mediated CaMKII/CREB Signaling Pathway in GABAergic Neurons of the Interpeduncular Nucleus
Source: Pharmaceuticals (Basel). 2025 Sep 28;18(10):1456. doi: 10.3390/ph18101456 (PMC12567535; doi:10.3390/ph18101456)
Supplement: Supplementary file 1 [file pharmaceuticals-18-01456-s001.zip › Table S2 Summary of Statistic.docx]

**Table S2. Summary of Statistic**

| **Experiment** | **Comparison** | **p-Value** |
| --- | --- | --- |
| von froy test (7d) | Sham vs. SNI | <0.0001 |
|  | Sham vs. STZYT (1.25g·kg^-1^) | 0.0002 |
|  | Sham vs. STZYT (0.625g·kg^-1^) | <0.0001 |
|  | Sham vs. Ifenprodil (6mg·kg^-1^) | <0.0001 |
| von froy test (14d) | SNI vs. STZYT (1.25g·kg^-1^) | 0.0266 |
|  | SNI vs. STZYT (0.625g·kg^-1^) | 0.1097 |
|  | SNI vs. Ifenprodil (6mg·kg^-1^) | 0.0007 |
| Hargreaves test (7d) | Sham vs. SNI | 0.0004 |
|  | Sham vs. STZYT (1.25g·kg^-1^) | 0.0001 |
|  | Sham vs. STZYT (0.625g·kg^-1^) | <0.0001 |
|  | Sham vs. Ifenprodil (6mg·kg^-1^) | <0.0001 |
| Hargreaves test (14d) | SNI vs. STZYT (1.25g·kg^-1^) | 0.0020 |
|  | SNI vs. STZYT (0.625g·kg^-1^) | 0.0017 |
|  | SNI vs. Ifenprodil (6mg·kg^-1^) | 0.0003 |
| Hot plate test (7d) | Sham vs. SNI | <0.0001 |
|  | Sham vs. STZYT (1.25g·kg^-1^) | <0.0001 |
|  | Sham vs. STZYT (0.625g·kg-1) | <0.0001 |
|  | Sham vs. Ifenprodil (6mg·kg^-1^) | <0.0001 |
| Hot plate test (14d) | SNI vs. STZYT (1.25g·kg^-1^) | 0.0077 |
|  | SNI vs. STZYT (0.625g·kg-1) | 0.0481 |
|  | SNI vs. Ifenprodil (6mg·kg^-1^) | 0.0029 |
| Light-dark box times of shuttle(7d) | Sham vs. SNI | <0.0001 |
|  | Sham vs. STZYD-H(1.25g·kg^-1^) | <0.0001 |
|  | Sham vs. STZYD-L (0.625g·kg^-1^) | 0.0010 |
|  | Sham vs. Ifenprodil (6mg·kg^-1^) | <0.0001 |
| Light-dark box times of shuttle(14d) | SNI vs. Ifenprodil (6mg·kg^-1^) | 0.0073 |
|  | SNI vs. STZYD-L (0.625g·kg^-1^) | 0.0115 |
|  | SNI vs. STZYD-H(1.25g·kg^-1^) | 0.0036 |
| Light box residence time(s) (7d) | Sham vs. SNI | 0.0221 |
|  | Sham vs. STZYD-H(1.25g·kg^-1^) | 0.0051 |
|  | Sham vs. STZYD-L (0.625g·kg^-1^) | 0.0330 |
|  | Sham vs. Ifenprodil (6mg·kg^-1^) | 0.0430 |
| Light box residence time(s) (14d) | SNI vs. STZYD-H(1.25g·kg^-1^) | 0.0279 |
|  | SNI vs. STZYD-L (0.625g·kg^-1^) | 0.0159 |
|  | SNI vs. Ifenprodil (6mg·kg^-1^) | 0.0002 |
| c-fos | Sham vs. SNI | <0.0001 |
|  | SNI vs. STZYT (0.625g·kg^-1^) | 0.0048 |
|  | SNI vs. STZYT (1.25g·kg^-1^) | 0.0019 |
|  | SNI vs. Ifenprodil (6mg·kg^-1^) | 0.0004 |
| AUC (von froy 0.4g) | post-SNI vs. pre-SNI | <0.0001 |
|  | post-STZYD-H vs. pre-STZYD-H | 0.0073 |
|  | post-STZYD-L vs. pre-STZYD-L | 0.1348 |
|  | post-ifenprodil vs. pre-ifenprodil | 0.0008 |
| Peak (von froy 0.4g) | post-SNI vs. pre-SNI | <0.0001 |
|  | post-STZYD-H vs. pre-STZYD-H | 0.0045 |
|  | post-STZYD-L vs. pre-STZYD-L | 0.2473 |
|  | post-ifenprodil vs. pre-ifenprodil | <0.0001 |
| AUC (Hargreaves test) | post-SNI vs. pre-SNI | 0.0014 |
|  | post-STZYD-H vs. pre-STZYD-H | 0.0006 |
|  | post-STZYD-L vs. pre-STZYD-L | 0.0293 |
|  | post-ifenprodil vs. pre-ifenprodil | 0.0002 |
| Peak (Hargreaves test) | post-SNI vs. pre-SNI | 0.0001 |
|  | post-STZYD-H vs. pre-STZYD-H | 0.0003 |
|  | post-STZYD-L vs. pre-STZYD-L | 0.0391 |
|  | post-ifenprodil vs. pre-ifenprodil | 0.0002 |
| hM4D(Gi) (von froy 0.4g) | 14d:hM4Di＋CNO vs. 1h:hM4Di＋CNO | 0.0149 |
|  | 1h:hM4Di＋CNO vs. 1h:hM4Di＋Saline | 0.0149 |
| hM4D(Gi) (Hargreaves test) | 14d:hM4Di＋CNO vs. 1h:hM4Di＋CNO | 0.0143 |
|  | 1h:hM4Di＋CNO vs. 1h:hM4Di＋Saline | 0.0414 |
| hM4D(Gq) (von froy 0.4g) | 14d:hM4Dq＋CNO vs. 1h:hM4Dq＋CNO | 0.0057 |
|  | 1h:hM4Dq＋CNO vs. 1h:hM4Dq＋Saline | 0.0101 |
| hM4D(Gq) (Hargreaves test) | 14d:hM4Dq＋CNO vs. 1h:hM4Dq＋CNO | 0.0110 |
|  | 1h:hM4Dq＋CNO vs. 1h:hM4Dq＋Saline | 0.0120 |
| GluN1 (relative mRNA expression) | Sham vs. SNI | 0.9014 |
|  | SNI vs. Ifenprodil (6mg·kg^-1^) | 0.9999 |
|  | SNI vs. STZYD-L (0.625g·kg^-1^) | 0.6034 |
|  | SNI vs. STZYD-H(1.25g·kg^-1^) | 0.3115 |
| GluN2A (relative mRNA expression) | Sham vs. SNI | 0.0484 |
|  | SNI vs. Ifenprodil (6mg·kg^-1^) | 0.1359 |
|  | SNI vs. STZYD-L (0.625g·kg^-1^) | 0.7304 |
|  | SNI vs. STZYD-H(1.25g·kg^-1^) | 0.4100 |
| GluN2B (relative mRNA expression) | Sham vs. SNI | 0.0308 |
|  | SNI vs. Ifenprodil (6mg·kg^-1^) | 0.0029 |
|  | SNI vs. STZYD-L (0.625g·kg^-1^) | 0.0365 |
|  | SNI vs. STZYD-H(1.25g·kg^-1^) | 0.0031 |
| GluA1(relative mRNA expression) | Sham vs. SNI | 0.6267 |
|  | SNI vs. Ifenprodil (6mg·kg^-1^) | 0.9992 |
|  | SNI vs. STZYD-L (0.625g·kg^-1^) | >0.9999 |
|  | SNI vs. STZYD-H(1.25g·kg^-1^) | 0.9969 |
| GluA2(relative mRNA expression) | Sham vs. SNI | 0.8930 |
|  | SNI vs. Ifenprodil (6mg·kg^-1^) | 0.8826 |
|  | SNI vs. STZYD-L (0.625g·kg^-1^) | 0.9859 |
|  | SNI vs. STZYD-H(1.25g·kg^-1^) | 0.0996 |
| GluA3(relative mRNA expression) | Sham vs. SNI | 0.5988 |
|  | SNI vs. Ifenprodil (6mg·kg^-1^) | 0.9497 |
|  | SNI vs. STZYD-L (0.625g·kg^-1^) | 0.7726 |
|  | SNI vs. STZYD-H(1.25g·kg^-1^) | >0.9999 |
| GluA4(relative mRNA expression) | Sham vs. SNI | 0.9875 |
|  | SNI vs. Ifenprodil (6mg·kg^-1^) | 0.7285 |
|  | SNI vs. STZYD-L (0.625g·kg^-1^) | 0.7616 |
|  | SNI vs. STZYD-H(1.25g·kg^-1^) | 0.9875 |
| Relative protein expression  of NMDAR2B/Vinculin | Sham vs. SNI | 0.0001 |
|  | SNI vs. STZYD-H(1.25g·kg^-1^) | <0.0001 |
|  | SNI vs. STZYD-L (0.625g·kg^-1^) | 0.0002 |
|  | SNI vs. Ifenprodil (6mg·kg^-1^) | <0.0001 |
| Relative protein expression  of p-CaMKII/CaMKII | Sham vs. SNI | 0.0002 |
|  | SNI vs. STZYD-H(1.25g·kg^-1^) | 0.0009 |
|  | SNI vs. STZYD-L (0.625g·kg^-1^) | 0.0027 |
|  | SNI vs. Ifenprodil (6mg·kg^-1^) | <0.0001 |
| Relative protein expression  of p-CREB/CREB | Sham vs. SNI | 0.0151 |
|  | SNI vs. STZYD-H(1.25g·kg^-1^) | 0.0002 |
|  | SNI vs. STZYD-L (0.625g·kg^-1^) | 0.0013 |
|  | SNI vs. Ifenprodil (6mg·kg^-1^) | <0.0001 |
| % of NMDAR-2B+ cells | Sham vs. SNI | <0.0001 |
|  | SNI vs. Ifenprodil (6mg·kg^-1^) | 0.0001 |
|  | SNI vs. STZYD-L (0.625g·kg^-1^) | 0.0208 |
|  | SNI vs. STZYD-H(1.25g·kg^-1^) | 0.0036 |
| % of p-CaMKII+ cells | Sham vs. SNI | <0.0001 |
|  | SNI vs. Ifenprodil (6mg·kg^-1^) | 0.0002 |
|  | SNI vs. STZYD-L (0.625g·kg^-1^) | 0.0889 |
|  | SNI vs. STZYD-H(1.25g·kg^-1^) | 0.0009 |
| % of p-CREB+ cells | Sham vs. SNI | <0.0001 |
|  | SNI vs. Ifenprodil (6mg·kg^-1^) | 0.0003 |
|  | SNI vs. STZYD-L (0.625g·kg^-1^) | 0.1179 |
|  | SNI vs. STZYD-H(1.25g·kg^-1^) | 0.0038 |
| Asp-(-120~-90min) | Sham vs. SNI | <0.0001 |
|  | SNI vs. STZYT (1.25g·kg^-1^) | 0.0011 |
|  | SNI vs. STZYT (0.625g·kg^-1^) | 0.8964 |
|  | SNI vs. Ifenprodil (6mg·kg^-1^) | <0.0001 |
| Asp-(-90~-60min) | Sham vs. SNI | <0.0001 |
|  | SNI vs. STZYT (1.25g·kg^-1^) | 0.0668 |
|  | SNI vs. STZYT (0.625g·kg^-1^) | 0.5944 |
|  | SNI vs. Ifenprodil (6mg·kg^-1^) | 0.0007 |
| Asp-(-60~-30min) | Sham vs. SNI | <0.0001 |
|  | SNI vs. STZYT (1.25g·kg^-1^) | 0.0533 |
|  | SNI vs. STZYT (0.625g·kg^-1^) | 0.7410 |
|  | SNI vs. Ifenprodil (6mg·kg^-1^) | <0.0001 |
| Asp-(-30~0min) | Sham vs. SNI | <0.0001 |
|  | SNI vs. STZYT (1.25g·kg^-1^) | 0.0030 |
|  | SNI vs. STZYT (0.625g·kg^-1^) | 0.2367 |
|  | SNI vs. Ifenprodil (6mg·kg^-1^) | <0.0001 |
| Asp-(0~30min) | Sham vs. SNI | <0.0001 |
|  | SNI vs. STZYT (1.25g·kg^-1^) | 0.0229 |
|  | SNI vs. STZYT (0.625g·kg^-1^) | 0.0760 |
|  | SNI vs. Ifenprodil (6mg·kg^-1^) | <0.0001 |
| Asp-(30~60min) | Sham vs. SNI | <0.0001 |
|  | SNI vs. STZYT (1.25g·kg^-1^) | 0.0002 |
|  | SNI vs. STZYT (0.625g·kg^-1^) | 0.0015 |
|  | SNI vs. Ifenprodil (6mg·kg^-1^) | <0.0001 |
| Asp-(60~90min) | Sham vs. SNI | <0.0001 |
|  | SNI vs. STZYT (1.25g·kg^-1^) | 0.0003 |
|  | SNI vs. STZYT (0.625g·kg^-1^) | 0.0298 |
|  | SNI vs. Ifenprodil (6mg·kg^-1^) | <0.0001 |
| Asp-(90~120min) | Sham vs. SNI | <0.0001 |
|  | SNI vs. STZYT (1.25g·kg^-1^) | 0.0461 |
|  | SNI vs. STZYT (0.625g·kg^-1^) | 0.6932 |
|  | SNI vs. Ifenprodil (6mg·kg^-1^) | 0.0068 |
| Asp-(120~150min) | Sham vs. SNI | <0.0001 |
|  | SNI vs. STZYT (1.25g·kg^-1^) | 0.0101 |
|  | SNI vs. STZYT (0.625g·kg^-1^) | 0.9454 |
|  | SNI vs. Ifenprodil (6mg·kg^-1^) | 0.0001 |
| Asp-(150~180min) | Sham vs. SNI | <0.0001 |
|  | SNI vs. STZYT (1.25g·kg^-1^) | 0.0144 |
|  | SNI vs. STZYT (0.625g·kg^-1^) | 0.1423 |
|  | SNI vs. Ifenprodil (6mg·kg^-1^) | <0.0001 |
| Asp-(180~210min) | Sham vs. SNI | <0.0001 |
|  | SNI vs. STZYT (1.25g·kg^-1^) | 0.5149 |
|  | SNI vs. STZYT (0.625g·kg^-1^) | 0.6909 |
|  | SNI vs. Ifenprodil (6mg·kg^-1^) | <0.0001 |
| Asp-(210~240min) | Sham vs. SNI | <0.0001 |
|  | SNI vs. STZYT (1.25g·kg^-1^) | 0.0620 |
|  | SNI vs. STZYT (0.625g·kg^-1^) | 0.5827 |
|  | SNI vs. Ifenprodil (6mg·kg^-1^) | <0.0001 |
| Glu-(-120~-90min) | Sham vs. SNI | <0.0001 |
|  | SNI vs. STZYT (1.25g·kg^-1^) | 0.1538 |
|  | SNI vs. STZYT (0.625g·kg^-1^) | 0.6881 |
|  | SNI vs. Ifenprodil (6mg·kg^-1^) | <0.0001 |
| Glu-(-90~-60min) | Sham vs. SNI | <0.0001 |
|  | SNI vs. STZYT (1.25g·kg^-1^) | 0.3017 |
|  | SNI vs. STZYT (0.625g·kg^-1^) | 0.9707 |
|  | SNI vs. Ifenprodil (6mg·kg^-1^) | <0.0001 |
| Glu -(-60~-30min) | Sham vs. SNI | <0.0001 |
|  | SNI vs. STZYT (1.25g·kg^-1^) | 0.4006 |
|  | SNI vs. STZYT (0.625g·kg^-1^) | 0.2207 |
|  | SNI vs. Ifenprodil (6mg·kg^-1^) | <0.0001 |
| Glu -(-30~0min) | Sham vs. SNI | <0.0001 |
|  | SNI vs. STZYT (1.25g·kg^-1^) | 0.0173 |
|  | SNI vs. STZYT (0.625g·kg^-1^) | 0.0157 |
|  | SNI vs. Ifenprodil (6mg·kg^-1^) | <0.0001 |
| Glu -(0~30min) | Sham vs. SNI | <0.0001 |
|  | SNI vs. STZYT (1.25g·kg^-1^) | 0.0045 |
|  | SNI vs. STZYT (0.625g·kg^-1^) | 0.2831 |
|  | SNI vs. Ifenprodil (6mg·kg^-1^) | <0.0001 |
| Glu -(30~60min) | Sham vs. SNI | <0.0001 |
|  | SNI vs. STZYT (1.25g·kg^-1^) | 0.0014 |
|  | SNI vs. STZYT (0.625g·kg^-1^) | 0.0317 |
|  | SNI vs. Ifenprodil (6mg·kg^-1^) | <0.0001 |
| Glu -(60~90min) | Sham vs. SNI | <0.0001 |
|  | SNI vs. STZYT (1.25g·kg^-1^) | 0.0002 |
|  | SNI vs. STZYT (0.625g·kg^-1^) | 0.0022 |
|  | SNI vs. Ifenprodil (6mg·kg^-1^) | <0.0001 |
| Glu -(90~120min) | Sham vs. SNI | <0.0001 |
|  | SNI vs. STZYT (1.25g·kg^-1^) | 0.0077 |
|  | SNI vs. STZYT (0.625g·kg^-1^) | 0.0033 |
|  | SNI vs. Ifenprodil (6mg·kg^-1^) | <0.0001 |
| Glu -(120~150min) | Sham vs. SNI | <0.0001 |
|  | SNI vs. STZYT (1.25g·kg^-1^) | 0.0033 |
|  | SNI vs. STZYT (0.625g·kg^-1^) | 0.8099 |
|  | SNI vs. Ifenprodil (6mg·kg^-1^) | <0.0001 |
| Glu -(150~180min) | Sham vs. SNI | <0.0001 |
|  | SNI vs. STZYT (1.25g·kg^-1^) | <0.0001 |
|  | SNI vs. STZYT (0.625g·kg^-1^) | 0.0019 |
|  | SNI vs. Ifenprodil (6mg·kg^-1^) | <0.0001 |
| Glu -(180~210min) | Sham vs. SNI | <0.0001 |
|  | SNI vs. STZYT (1.25g·kg^-1^) | 0.0466 |
|  | SNI vs. STZYT (0.625g·kg^-1^) | 0.0051 |
|  | SNI vs. Ifenprodil (6mg·kg^-1^) | <0.0001 |
| Glu -(210~240min) | Sham vs. SNI | <0.0001 |
|  | SNI vs. STZYT (1.25g·kg^-1^) | 0.0024 |
|  | SNI vs. STZYT (0.625g·kg^-1^) | 0.4474 |
|  | SNI vs. Ifenprodil (6mg·kg^-1^) | <0.0001 |
| Ser -(-120~-90min) | Sham vs. SNI | 0.0035 |
|  | SNI vs. STZYT (1.25g·kg^-1^) | 0.1300 |
|  | SNI vs. STZYT (0.625g·kg^-1^) | 0.1002 |
|  | SNI vs. Ifenprodil (6mg·kg^-1^) | 0.0422 |
| Ser -(-90~-60min) | Sham vs. SNI | 0.0011 |
|  | SNI vs. STZYT (1.25g·kg^-1^) | 0.0836 |
|  | SNI vs. STZYT (0.625g·kg^-1^) | 0.7083 |
|  | SNI vs. Ifenprodil (6mg·kg^-1^) | 0.0275 |
| Ser -(-60~-30min) | Sham vs. SNI | 0.0001 |
|  | SNI vs. STZYT (1.25g·kg^-1^) | 0.2411 |
|  | SNI vs. STZYT (0.625g·kg^-1^) | 0.6138 |
|  | SNI vs. Ifenprodil (6mg·kg^-1^) | 0.1002 |
| Ser -(-30~0min) | Sham vs. SNI | 0.0003 |
|  | SNI vs. STZYT (1.25g·kg^-1^) | 0.2765 |
|  | SNI vs. STZYT (0.625g·kg^-1^) | 0.2765 |
|  | SNI vs. Ifenprodil (6mg·kg^-1^) | 0.0045 |
| Ser -(0~30min) | Sham vs. SNI | 0.1095 |
|  | SNI vs. STZYT (1.25g·kg^-1^) | 0.6379 |
|  | SNI vs. STZYT (0.625g·kg^-1^) | 0.9233 |
|  | SNI vs. Ifenprodil (6mg·kg^-1^) | 0.3996 |
| Ser -(30~60min) | Sham vs. SNI | 0.0035 |
|  | SNI vs. STZYT (1.25g·kg^-1^) | 0.3148 |
|  | SNI vs. STZYT (0.625g·kg^-1^) | 0.3148 |
|  | SNI vs. Ifenprodil (6mg·kg^-1^) | 0.0517 |
| Ser -(60~90min) | Sham vs. SNI | 0.0076 |
|  | SNI vs. STZYT (1.25g·kg^-1^) | 0.2953 |
|  | SNI vs. STZYT (0.625g·kg^-1^) | 0.4453 |
|  | SNI vs. Ifenprodil (6mg·kg^-1^) | 0.0247 |
| Ser -(90~120min) | Sham vs. SNI | 0.0002 |
|  | SNI vs. STZYT (1.25g·kg^-1^) | 0.0221 |
|  | SNI vs. STZYT (0.625g·kg^-1^) | 0.0422 |
|  | SNI vs. Ifenprodil (6mg·kg^-1^) | 0.0006 |
| Ser -(120~150min) | Sham vs. SNI | 0.0027 |
|  | SNI vs. STZYT (1.25g·kg^-1^) | 0.0052 |
|  | SNI vs. STZYT (0.625g·kg^-1^) | 0.0380 |
|  | SNI vs. Ifenprodil (6mg·kg^-1^) | 0.0139 |
| Ser -(150~180min) | Sham vs. SNI | 0.0110 |
|  | SNI vs. STZYT (1.25g·kg^-1^) | 0.0517 |
|  | SNI vs. STZYT (0.625g·kg^-1^) | 0.7944 |
|  | SNI vs. Ifenprodil (6mg·kg^-1^) | 0.2584 |
| Ser -(180~210min) | Sham vs. SNI | 0.0067 |
|  | SNI vs. STZYT (1.25g·kg^-1^) | 0.4925 |
|  | SNI vs. STZYT (0.625g·kg^-1^) | 0.7944 |
|  | SNI vs. Ifenprodil (6mg·kg^-1^) | 0.5895 |
| Ser -(210~240min) | Sham vs. SNI | 0.0275 |
|  | SNI vs. STZYT (1.25g·kg^-1^) | 0.1002 |
|  | SNI vs. STZYT (0.625g·kg^-1^) | 0.6852 |
|  | SNI vs. Ifenprodil (6mg·kg^-1^) | 0.0836 |
| Gly-(-120~-90min) | Sham vs. SNI | <0.0001 |
|  | SNI vs. STZYT (1.25g·kg^-1^) | 0.0034 |
|  | SNI vs. STZYT (0.625g·kg^-1^) | 0.2449 |
|  | SNI vs. Ifenprodil (6mg·kg^-1^) | <0.0001 |
| Gly -(-90~-60min) | Sham vs. SNI | <0.0001 |
|  | SNI vs. STZYT (1.25g·kg^-1^) | 0.0119 |
|  | SNI vs. STZYT (0.625g·kg^-1^) | 0.4774 |
|  | SNI vs. Ifenprodil (6mg·kg^-1^) | <0.0001 |
| Gly -(-60~-30min) | Sham vs. SNI | <0.0001 |
|  | SNI vs. STZYT (1.25g·kg^-1^) | 0.0264 |
|  | SNI vs. STZYT (0.625g·kg^-1^) | 0.3622 |
|  | SNI vs. Ifenprodil (6mg·kg^-1^) | 0.0003 |
| Gly -(-30~0min) | Sham vs. SNI | <0.0001 |
|  | SNI vs. STZYT (1.25g·kg^-1^) | 0.0076 |
|  | SNI vs. STZYT (0.625g·kg^-1^) | 0.1680 |
|  | SNI vs. Ifenprodil (6mg·kg^-1^) | <0.0001 |
| Gly -(0~30min) | Sham vs. SNI | <0.0001 |
|  | SNI vs. STZYT (1.25g·kg^-1^) | 0.0121 |
|  | SNI vs. STZYT (0.625g·kg^-1^) | 0.1843 |
|  | SNI vs. Ifenprodil (6mg·kg^-1^) | <0.0001 |
| Gly -(30~60min) | Sham vs. SNI | <0.0001 |
|  | SNI vs. STZYT (1.25g·kg^-1^) | <0.0001 |
|  | SNI vs. STZYT (0.625g·kg^-1^) | 0.0010 |
|  | SNI vs. Ifenprodil (6mg·kg^-1^) | <0.0001 |
| Gly -(60~90min) | Sham vs. SNI | <0.0001 |
|  | SNI vs. STZYT (1.25g·kg^-1^) | 0.0001 |
|  | SNI vs. STZYT (0.625g·kg^-1^) | 0.0004 |
|  | SNI vs. Ifenprodil (6mg·kg^-1^) | <0.0001 |
| Gly -(90~120min) | Sham vs. SNI | <0.0001 |
|  | SNI vs. STZYT (1.25g·kg^-1^) | 0.0131 |
|  | SNI vs. STZYT (0.625g·kg^-1^) | 0.0943 |
|  | SNI vs. Ifenprodil (6mg·kg^-1^) | 0.0054 |
| Gly -(120~150min) | Sham vs. SNI | <0.0001 |
|  | SNI vs. STZYT (1.25g·kg^-1^) | 0.0508 |
|  | SNI vs. STZYT (0.625g·kg^-1^) | 0.7263 |
|  | SNI vs. Ifenprodil (6mg·kg^-1^) | 0.0002 |
| Gly -(150~180min) | Sham vs. SNI | <0.0001 |
|  | SNI vs. STZYT (1.25g·kg^-1^) | 0.0722 |
|  | SNI vs. STZYT (0.625g·kg^-1^) | 0.3155 |
|  | SNI vs. Ifenprodil (6mg·kg^-1^) | <0.0001 |
| Gly -(180~210min) | Sham vs. SNI | <0.0001 |
|  | SNI vs. STZYT (1.25g·kg^-1^) | 0.2560 |
|  | SNI vs. STZYT (0.625g·kg^-1^) | 0.2437 |
|  | SNI vs. Ifenprodil (6mg·kg^-1^) | 0.0004 |
| Gly -(210~240min) | Sham vs. SNI | <0.0001 |
|  | SNI vs. STZYT (1.25g·kg^-1^) | 0.1130 |
|  | SNI vs. STZYT (0.625g·kg^-1^) | 0.2976 |
|  | SNI vs. Ifenprodil (6mg·kg^-1^) | 0.0010 |
| GABA-(-120~-90min) | Sham vs. SNI | <0.0001 |
|  | SNI vs. STZYT (1.25g·kg^-1^) | 0.0003 |
|  | SNI vs. STZYT (0.625g·kg^-1^) | 0.0018 |
|  | SNI vs. Ifenprodil (6mg·kg^-1^) | <0.0001 |
| GABA -(-90~-60min) | Sham vs. SNI | <0.0001 |
|  | SNI vs. STZYT (1.25g·kg^-1^) | <0.0001 |
|  | SNI vs. STZYT (0.625g·kg^-1^) | 0.0007 |
|  | SNI vs. Ifenprodil (6mg·kg^-1^) | <0.0001 |
| GABA -(-60~-30min) | Sham vs. SNI | <0.0001 |
|  | SNI vs. STZYT (1.25g·kg^-1^) | 0.0002 |
|  | SNI vs. STZYT (0.625g·kg^-1^) | 0.0019 |
|  | SNI vs. Ifenprodil (6mg·kg^-1^) | <0.0001 |
| GABA-(-30~0min) | Sham vs. SNI | <0.0001 |
|  | SNI vs. STZYT (1.25g·kg^-1^) | 0.0006 |
|  | SNI vs. STZYT (0.625g·kg^-1^) | 0.0047 |
|  | SNI vs. Ifenprodil (6mg·kg^-1^) | <0.0001 |
| GABA -(0~30min) | Sham vs. SNI | <0.0001 |
|  | SNI vs. STZYT (1.25g·kg^-1^) | 0.0020 |
|  | SNI vs. STZYT (0.625g·kg^-1^) | 0.0245 |
|  | SNI vs. Ifenprodil (6mg·kg^-1^) | <0.0001 |
| GABA -(30~60min) | Sham vs. SNI | <0.0001 |
|  | SNI vs. STZYT (1.25g·kg^-1^) | <0.0001 |
|  | SNI vs. STZYT (0.625g·kg^-1^) | <0.0001 |
|  | SNI vs. Ifenprodil (6mg·kg^-1^) | <0.0001 |
| GABA -(60~90min) | Sham vs. SNI | <0.0001 |
|  | SNI vs. STZYT (1.25g·kg^-1^) | <0.0001 |
|  | SNI vs. STZYT (0.625g·kg^-1^) | <0.0001 |
|  | SNI vs. Ifenprodil (6mg·kg^-1^) | <0.0001 |
| GABA -(90~120min) | Sham vs. SNI | <0.0001 |
|  | SNI vs. STZYT (1.25g·kg^-1^) | <0.0001 |
|  | SNI vs. STZYT (0.625g·kg^-1^) | <0.0001 |
|  | SNI vs. Ifenprodil (6mg·kg^-1^) | <0.0001 |
| GABA -(120~150min) | Sham vs. SNI | <0.0001 |
|  | SNI vs. STZYT (1.25g·kg^-1^) | <0.0001 |
|  | SNI vs. STZYT (0.625g·kg^-1^) | 0.0004 |
|  | SNI vs. Ifenprodil (6mg·kg^-1^) | <0.0001 |
| GABA -(150~180min) | Sham vs. SNI | <0.0001 |
|  | SNI vs. STZYT (1.25g·kg^-1^) | 0.0007 |
|  | SNI vs. STZYT (0.625g·kg^-1^) | 0.0005 |
|  | SNI vs. Ifenprodil (6mg·kg^-1^) | <0.0001 |
| GABA -(180~210min) | Sham vs. SNI | <0.0001 |
|  | SNI vs. STZYT (1.25g·kg^-1^) | <0.0001 |
|  | SNI vs. STZYT (0.625g·kg^-1^) | 0.0004 |
|  | SNI vs. Ifenprodil (6mg·kg^-1^) | <0.0001 |
| GABA -(210~240min) | Sham vs. SNI | <0.0001 |
|  | SNI vs. STZYT (1.25g·kg^-1^) | <0.0001 |
|  | SNI vs. STZYT (0.625g·kg^-1^) | 0.0014 |
|  | SNI vs. Ifenprodil (6mg·kg^-1^) | <0.0001 |
