# Supplementary figures and images for: Shentong Zhuyu Decoction Alleviates Neuropathic Pain in Mice by Inhibiting the NMDAR-2B Receptor-Mediated CaMKII/CREB Signaling Pathway in GABAergic Neurons of the Interpeduncular Nucleus
Source: Pharmaceuticals (Basel). 2025 Sep 28;18(10):1456. doi: 10.3390/ph18101456 (PMC12567535; doi:10.3390/ph18101456)

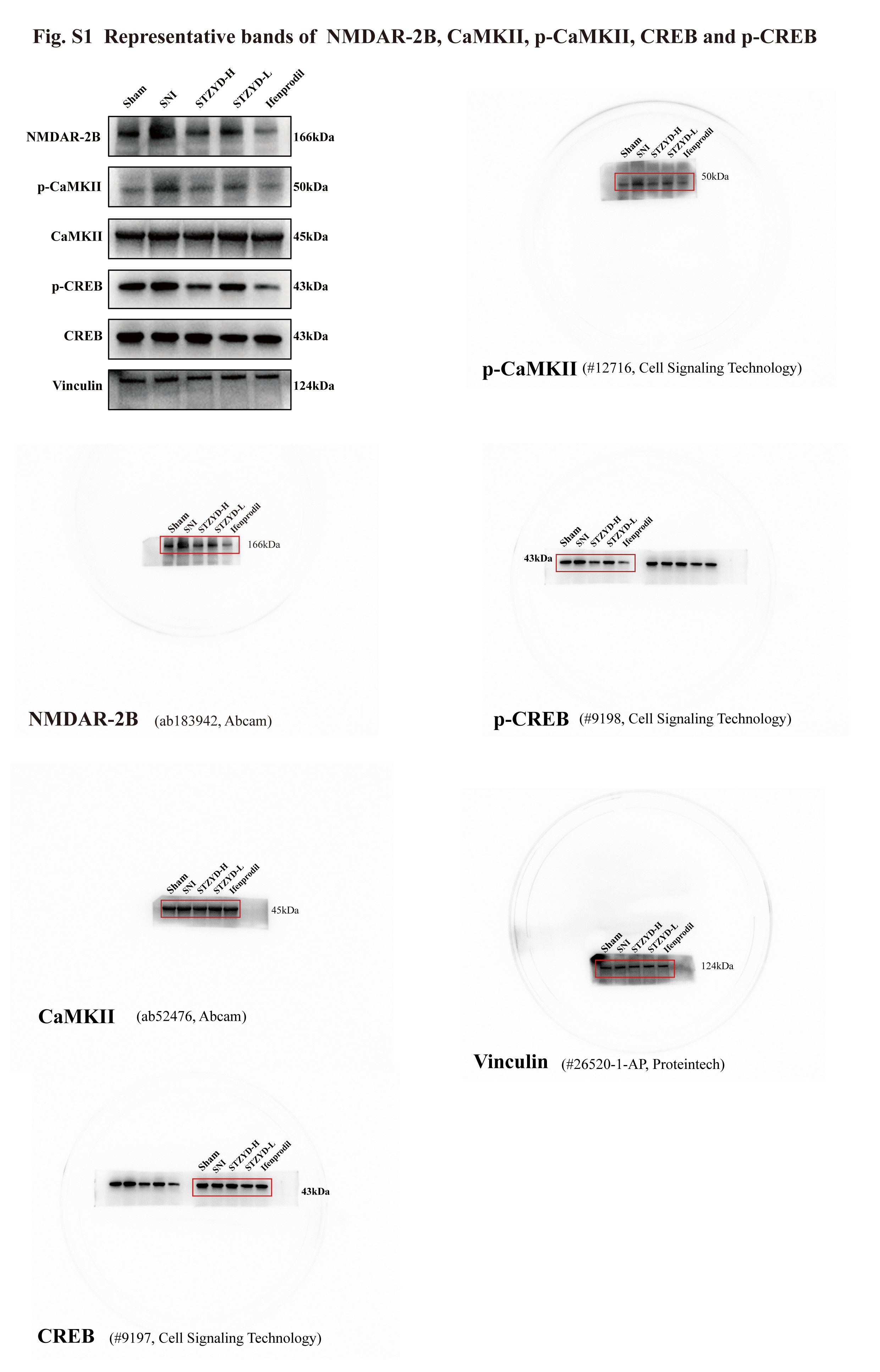

Supplement: Supplementary file 1 [file pharmaceuticals-18-01456-s001.zip › FIgure S1.tif]
